# Unraveling Subcellular and Ultrastructural Changes During Vitrification of Human Spermatozoa: Effect of a Mitochondria-Targeted Antioxidant and a Permeable Cryoprotectant

**DOI:** 10.3389/fcell.2021.672862

**Published:** 2021-07-02

**Authors:** Pradeep Kumar, Mengying Wang, Evgenia Isachenko, Gohar Rahimi, Peter Mallmann, Wanxue Wang, Melanie von Brandenstein, Vladimir Isachenko

**Affiliations:** ^1^Department of Animal Physiology and Reproduction, ICAR-Central Institute for Research on Buffaloes, Hisar, India; ^2^Department of Obstetrics and Gynaecology, Medical Faculty, Cologne University, Cologne, Germany; ^3^Department of Urology, University Hospital of Cologne, Cologne, Germany

**Keywords:** MitoQ, vitrification, proteomics, electron microscopy, spermatozoa

## Abstract

Mitochondria-targeted antioxidants have great potential to counterbalance the generated reactive oxygen species (ROS) because they cross the inner membrane of the mitochondria. Still, their use was not reported in vitrified human spermatozoa. Our laboratory has successfully vitrified spermatozoa without the use of permeable cryoprotectants, but subcellular-level evidence was missing. Therefore, this study aimed to improve spermatozoa vitrification using a mitochondria-targeted antioxidant (mitoquinone, MitoQ), reveal ultrastructural changes in the spermatozoa due to the use of a permeable cryoprotectant, and report alterations of functional proteins during the spermatozoa vitrification process. For this, each of 20 swim-up-prepared ejaculates was divided into seven aliquots and diluted with a vitrification medium supplemented with varying concentrations of MitoQ (0.02 and 0.2 μM), glycerol (1, 4, and 6%), and a combination of MitoQ and glycerol. All aliquots were vitrified by the aseptic capillary method developed in our laboratory. The spermatozoa function assays revealed that the addition of either MitoQ (0.02 μM), glycerol (1%), or a combination of MitoQ (0.02 μM) and glycerol (1%) in the vitrification medium results in better or equivalent spermatozoa quality relative to the control. Transmission electron microscopy revealed that MitoQ protects the spermatozoa from undergoing ultrastructural alterations, but glycerol induced ultrastructural alterations during the vitrification process. Next, we performed label-free quantitative proteomics and identified 1,759 proteins, of which 69, 60, 90, and 81 were altered in the basal medium, 0.02 μM MitoQ, 1% glycerol, and Mito-glycerol groups, respectively. Actin, tubulins, and outer dense fiber proteins were not affected during the vitrification process. Some of the identified ubiquitinating enzymes were affected during spermatozoa vitrification. Only a few proteins responsible for phosphorylation were altered during vitrification. Similarly, several proteins involved in spermatozoa–egg fusion and fertilization (IZUMO1 and Tektin) were not affected during the vitrification process. In conclusion, MitoQ attenuates the vitrification-induced ultrastructural changes and alterations in the key proteins involved in spermatozoa functions and fertilization.

## Introduction

Spermatozoa vitrification was accomplished by plunging small volumes of spermatozoa into liquid nitrogen; the technique has been termed “kinetic vitrification” because of the ultra-rapid cooling rates. Kinetic vitrification for spermatozoa is different from a conventional term for vitrification linked with oocytes and embryos (Katkov et al., 2006). For the first time, we successfully established the vitrification of human spermatozoa without permeable cryoprotectants (Nawroth et al., 2002; Isachenko et al., 2004, 2008). We developed aseptic technology for the vitrification of human spermatozoa in a large volume (0.5 ml) for intrauterine insemination (Isachenko et al., 2011) and in capillaries for intracytoplasmic spermatozoa injection or *in vitro* fertilization (Isachenko et al., 2012b). We reported the birth of two babies after intracytoplasmic spermatozoa injection using vitrified spermatozoa (Isachenko et al., 2012a) and a baby born after intrauterine insemination with vitrified spermatozoa from an oligoasthenozoospermia man (Sanchez et al., 2012).

During cryopreservation, the cryo-injured mitochondria start to produce reactive oxygen species (ROS) in an unchecked manner, causing oxidative damage to the spermatozoa (Thomson et al., 2009). To prevent ROS generation during cryopreservation, most of the antioxidants used as additives in the semen dilutor are not permeable to the mitochondrial inner membrane. In recent years, great advancement has been made in developing molecules that specifically target the mitochondria and protect cells from oxidative stress (Apostolova and Victor, 2015). A breakthrough in mitochondria-targeted antioxidants was reported in the late 1990s with the discovery of mitoquinone (MitoQ) (Murphy, 1997). MitoQ was synthesized by attaching lipophilic triphenylphosphonium (TPP) cation to the coenzyme Q10 (ubiquinone) (Smith et al., 1999). This passes into the mitochondria 800 to 1,200 times more than exogenous CoQ10. CoQ10 or ubiquinone passages electrons from complexes I and II to complex III in the mitochondrial respiratory chain (Carocho and Ferreira, 2013) and protects the mitochondria from oxidative damage due to its powerful antioxidant activity (Alleva et al., 1997). Therefore, our hypothesis is that supplementation of MitoQ in the vitrification medium might ameliorate the mitochondrial functionality in the vitrified spermatozoa.

The key cause of low fertility appears to be most related to the acrosomal integrity status (Nagae et al., 1986), and mitochondrial functionality is necessary to maintain the acrosome integrity of the spermatozoa (Isachenko et al., 2008; Zhang et al., 2019). The dramatic decline in the percentage of intact spermatozoa during cryopreservation is demonstrable using transmission electron microscopy (TEM) rather than conventional staining methods (Barthelemy et al., 1990). Therefore, we attempted to estimate the damage caused by vitrification using TEM. Also, subcellular reasons—like changes in the functional proteins—associated with the fertilization failure of the spermatozoa cannot be clearly explained with conventional semen analysis (Panner Selvam et al., 2019). The damage of spermatozoa proteins during spermatozoa cryopreservation undesirably affects fertilization and early embryonic development (Wang et al., 2014; Bogle et al., 2017; Gholami et al., 2018; Li et al., 2019). Recently, spermatozoa vitrification has been established as an alternative to the slow-freezing method of human spermatozoa. The protein alteration at the proteomics level during spermatozoa vitrification might help in the identification of a better spermatozoa vitrification protocol. We established a permeable cryoprotectant-free spermatozoa vitrification for human spermatozoa. However, a few studies have used the permeable cryoprotectant glycerol for human spermatozoa vitrification, resulting in controversial results (Saritha and Bongso, 2001; Vutyavanich et al., 2010; Le et al., 2019). Unfortunately, to our knowledge, no study has conducted proteomics of fresh and vitrified spermatozoa to determine the alterations to proteins during vitrification. Therefore, we planned for a proteomics study to identify subcellular changes after vitrification in our basic vitrification medium supplemented with the permeable cryoprotectant glycerol and the mitochondrial-targeted antioxidant MitoQ.

The aims of the present study were: (1) to investigate the effect of the mitochondria-targeted antioxidant MitoQ and the permeable cryoprotectant glycerol on ameliorating the spermatozoa vitrification process and (2) to determine ultrastructural changes and alterations of functional proteins after vitrification using TEM and label-free quantitative proteomics techniques.

## Materials and Methods

This study was approved by the Ethics Boards of the University of Cologne (application 01-106). Written informed consent was obtained from each participant involved in our study. Except where otherwise stated, all chemicals were obtained from Sigma (Sigma Chemical Co., St. Louis, MO, United States).

### Sperm Preparation

Ejaculates containing a minimum of 20 × 10^6^ sperm/ml and displaying 70–75% progressive sperm motility were collected from 20 healthy men by masturbation, after a minimum of 48 h of sexual abstinence. After liquefaction, each ejaculate was prepared by the swim-up method and divided into seven aliquots. Swim-up was performed using a culture medium [human tubal fluid (HTF); Irvine Scientific, Santa Ana, CA, United States] (Quinn et al., 1985) supplemented with 10 mg/ml of human serum albumin (HSA; Irvine). The swim-up-prepared sperm suspension was diluted (1:1) with 0.5 M sucrose at room temperature. The sperm suspension was divided into seven aliquots and supplemented with different concentrations of MitoQ and glycerol, as described below. The concentrations of MitoQ and glycerol were decided based on sperm tolerance to sperm motility during 24 h incubation of the swim-up-prepared sperm:

Control: Swim-up-prepared fresh sperm.
Treatment 1 (BM): basal medium (basic culture medium and 0.25 M sucrose).
Treatment 2 (0.02 μM MitoQ): basal medium supplemented with 0.02 μM MitoQ.
Treatment 3 (0.2 μM MitoQ): basal medium supplemented with 0.2 μM MitoQ.
Treatment 4 (1% glycerol): basal medium supplemented with 1% glycerol.
Treatment 5 (4% glycerol): basal medium supplemented with 4% glycerol.
Treatment 6 (6% glycerol): basal medium supplemented with 6% glycerol.
Treatment 7 (Mito-glycerol): basal medium supplemented with 0.02 μM MitoQ and 1% glycerol.

### Vitrification and Warming

Ten microliters spermatozoa suspension of each treatment group was aspirated in hydrophobic plastic capillaries (Gynemed GmbH & Co. KG, Lensahn, Germany). The capillary was first loaded into 0.25 ml straw (Medical Technology GmbH, Bruckberg, Germany), of which one side was already closed. After sealing the second side, the straw was plunged into liquid nitrogen. After 24 h, the upper part of the straw was cut and warming was performed by submerging a capillary in 2 ml of pre-warmed HTF-HSA at 42°C for 20 s. Finally, the suspension of spermatozoa was ejected from the capillary for an immediate evaluation of sperm quality. The following parameters were evaluated in the control and in each treatment group to determine the effect of MitoQ and glycerol supplementation on sperm quality.

### Sperm Motility

The sperm motility of the swim-up-prepared fresh sperm and each treatment group were estimated using the Makler chamber under a phase-contrast microscope at × 200 magnification. Sperm motility was categorized as progressively motile, non-progressively motile, and immotile (instead of grades a, b, c, or d) as per the latest edition (fifth) of the WHO laboratory manual for the examination and processing of human semen. The spermatozoon that moved actively, either linearly or in a large circle, regardless of speed, was considered progressive motile, while all other patterns of motility with an absence of progression, e.g., swimming in small circles, the flagellar force hardly displacing the head, or displaying only a flagellar beat, were considered as non-progressively motile (WHO, 2010). In the present study, the total sperm motility was recorded as progressive as well as non-progressive sperm motility.

### Sperm Viability

The live and dead sperm were evaluated using a live/dead sperm viability kit (#L7011, Molecular Probes, Eugene, OR, United States) as per the manufacturer’s instructions. Briefly, 20 μl spermatozoa suspension (about 20 × 10^6^/ml) of the swim-up-prepared fresh sperm and the vitrified sperm of each treatment group was incubated with SYBR-14 (1 μM) and propidium iodide (PI, 120 μM) in an Eppendorf tube at 37°C for 10 min. A 10-μl drop of the mix was placed on a slide under coverslips and evaluated under epifluorescence using Zeiss Axioskop epifluorescence microscope filter I (excitation filter, 450–490 nm; dichroic mirror, 510 nm; and emission filter, 515–565 nm) at × 630 magnification. Both live (green) and dead (red) sperm were counted simultaneously. About 200 sperm cells were counted on each slide. Each determination was carried out in duplicate.

### Acrosome Integrity

The acrosome damages during vitrification and warming were assessed by *Pisum sativum* agglutinin lectin (PSA) conjugated to fluorescein isothiocyanate (FITC). Briefly, 20 μl of sperm suspension (about 20 × 10^6^/ml) of the swim-up-prepared fresh sperm and the vitrified sperm of each treatment group was washed with Dulbecco’s phosphate-buffered saline (DPBS; #14190-094, Gibco, Waltham, MA, United States) by centrifugation to remove semen diluents; then, the pellet was resuspended in PBS. The sperm suspension was smeared onto a slide and fixed by cold 4% paraformaldehyde for 30 min at room temperature. The PSA–FITC solution (0.1 mg/ml; #L0770, Sigma-Aldrich, Steinheim, Germany) was placed over the smear and incubated in a humidified chamber at 37°C for 20 min, rinsed with PBS, and dried. The slides were examined under Zeiss Axioskop epifluorescence microscope filter I (excitation filter, 450–490 nm; dichroic mirror, 510 nm; and emission filter, 515–565 nm) with a × 100 oil objective. About 200 cells were counted on each slide. Each determination was carried out in duplicate. The bright fluorescence with a clear-cut acrosomal cap in the acrosomal region was considered an intact acrosome, while no fluorescence or only fluorescence of the equatorial segment or an irregular acrosomal cap was considered a damaged acrosome.

### Mitochondrial Membrane Potential

The mitochondrial membrane potential of the swim-up-prepared fresh sperm and the vitrified sperm of each treatment group was assessed by the tetramethylrhodamine (TMRM) staining method. To the prepared stock solution, TMRM (Sigma-Aldrich Inc., St Louis, MO, United States) was dissolved in dimethyl sulfoxide (DMSO) to make a 100-μM solution. The working solution was prepared from stock solution by dissolving in DPBS to make a 300-nM solution. The washed sperm were incubated in TMRM working solution for 30 min at 37°C. After incubation, the sperm suspension was washed with DPBS. Finally, the pellet was resuspended in DPBS and observed under Zeiss Axioskop epifluorescence microscope filter IV (excitation filter, 546 nm; dichroic mirror, 580 nm; and emission filter, 590 nm) with a × 100 oil objective. The sperm images were categorized into high and low mitochondrial potential based on the intensity of fluorescence of the mid-piece region of sperm. As many as 200 cells were counted on each slide in a blind fashion. Each determination was carried out in duplicate.

### DNA Fragmentation

The DNA fragmentation of the swim-up-prepared fresh sperm and the vitrified sperm of each treatment group was measured by the In Situ Cell Death Detection, Fluorescein kit (Roche Diagnostics, Rotkreuz, Switzerland) according to the manufacturer’s instructions, with slight modifications. Briefly, the air-dried sperm smear on a glass slide was fixed with a freshly prepared fixative solution (4% paraformaldehyde in DPBS, pH 7.4) for 1 h at room temperature and rinsed with PBS. After fixation, the sperm cells were permeabilized using Triton (0.1% in 0.1% sodium citrate) for 2 min at 4°C and washed with PBS. After this, the terminal deoxynucleotidyl transferase dUTP nick-end labeling (TUNEL) reaction mixture (containing terminal deoxynucleotidyl transferase and nucleotide) was poured on the dried slide, incubated in a humidified atmosphere for 60 min at 37°C in the dark, and washed with DPBS. The slides were examined under Zeiss Axioskop epifluorescence microscope filter I (excitation filter, 450–490 nm; dichroic mirror, 510 nm; and emission filter, 515–565 nm) with a × 100 oil objective. As many as 200 cells were counted on each slide in a blind fashion. The light green sperm head was considered TUNEL-negative, i.e., intact DNA, and the bright green sperm head was considered TUNEL-positive, i.e., damaged DNA.

### Determination of Intracellular ATP

ATP in the swim-up-prepared fresh sperm and the vitrified sperm of each treatment group was measured using the ATP bioluminescence assay kit CLS II (#11699695001, Roche) following the manufacturer’s instructions. Briefly, 50 μl washed sperm suspension (∼5 million sperm/ml) was mixed with 450 μl of boiling cell lysis reagent (100 mM Tris, 4 mM EDTA, pH 7.75) and incubated for another 2 min at 100°C. The lysed sperm suspension was centrifuged at 1,000 × *g* for 60 s and the supernatant collected on ice. The ATP standard was serially diluted with water in a range from 10^–5^ to 10^–10^ M of ATP. In each well, 50 μl of luciferase reagent and 50 μl of the sample/standard were added; following a 1-s delay, light emission was measured over a 10-s integration period using a luminometer (MPL1, Berthold Detection System, Pforzheim, Germany). A standard curve was constructed using solutions containing known concentrations of ATP, and the sample’s ATP was expressed as micromolar ATP/million sperm.

### CellROX Deep Red Assay

The CellROX Deep Red Reagent fluorescent probe (2.5 mM in DMSO; Life Technologies, New York, NY, United States) was used to measure the ROS in the swim-up-prepared fresh sperm and the vitrified sperm of each treatment group. Briefly, CellROX Deep Red Reagent (2.5 mM) was added to 30 μl sperm suspension (20 million sperm/ml) to obtain the final concentration of fluorescent probe of 5 μM in sperm suspension and incubated for 30 min at 37°C. After incubation, each sample was washed twice with DPBS by centrifuging for 5 min at 2,000 × *g*. The supernatant was removed and the pellet was resuspended in 30 μl of DPBS. An aliquot of 10 μl of the solution stained with CellROX^®^ and 10 μl of Fluoromount-G with DAPI (Invitrogen) was placed between a slide and a coverslip and read under an epifluorescence microscope (Zeiss Axioskop) at × 1,000 magnification using an excitation filter of 546 nm, dichroic mirror of 580 nm, and emission filter of 590 nm. The sperm was categorized into low (middle piece stained pale red or no color) and high (middle piece stained intense red) oxidative stress based on the intensity of fluorescence of the mid-piece region of sperm. As many as 200 cells were counted on each slide in a blind fashion. Each determination was carried out in duplicate.

### Transmission Electron Microscopy

To analyze ultrastructural changes in the vitrified sperm, only treatment groups BM, 0.02 μM MitoQ, 1% glycerol, and Mito-glycerol were selected based on the better results of conventional semen evaluation parameters (viability, motility, and acrosomal integrity) and compared with the swim-up-prepared sperm. The swim-up-prepared fresh sperm and the vitrified sperm of each treatment group were fixed for 48 h. The primary fixative was composed of 2% formaldehyde, 2% glutaraldehyde, and 3 mM CaCl_2_ in 0.1 M sodium cacodylate buffer (Applichem, Damstadt, Germany) for 48 h. Samples were washed with 0.1 M sodium cacodylate buffer, embedded into 1.5% low melting agarose, and cut into 1-mm^3^ cubes. Post-fixation was applied using 2% osmium tetroxide (Science Services, Munich, Germany) and 1% potassium ferrocyanide in 0.1 M cacodylate buffer for 2 h at 4°C. Samples were washed four times with 0.1 M cacodylate buffer and dehydrated using an ascending ethanol series (50%, 70%, 90%, and 3 × 100%) for 15 min each. The samples were incubated with a mix of 50% ethanol/propylene oxide and two times with pure propylene oxide for 15 min each step. Next, the samples were infiltrated with a mixture of 50% epon/propylene oxide and 75% epon/propylenoxide for 2 h each at 4°C and with pure epon overnight at 4°C. The next day, epon was exchanged and the samples were incubated for 2 h at room temperature, placed into TAAB capsules, and cured for 72 h at 60°C. Ultrathin sections of 70 nm were cut using an ultramicrotome (UC6, Leica Microsystems, Wetzlar, Germany) and a diamond knife (Diatome, Biel, Switzerland) and stained with 1.5% uranyl acetate for 15 min at 37°C and with Reynolds lead citrate solution for 4 min. Images were acquired using a JEM-2100 Plus transmission electron microscope (JEOL, Tokyo, Japan) operating at 80 kV equipped with a OneView 4K camera (Gatan, Pleasanton, CA, United States). The number of spermatozoa in each group was determined in a sample of 100 spermatozoa from at least two sets of sections. The sperm heads were classified as described by Mahadevan and Trounson (1984), with slight modifications. Type 1 consisted of sperm with undamaged, normal heads with an intact plasma membrane (PM), outer acrosomal (OA) membrane, acrosomal material (AM), inner acrosomal (IA) membrane, nuclear membrane (NM), and a nucleus (N). Type 2 consisted of sperm like those of type 1, except that the PM was swollen or damaged. Type 3 was like type 2, except that the acrosome was swollen and wavy but the AM appeared homogenous and normal (Figures 3C,D). In type 4, the OA and IA membranes were damaged and the AM had leaked out, dispersed, or condensed (Figures 3C,F). In type 5, the acrosome and PM were detached and appeared without a cap. In type 6, the IA membrane was absent and the NM could also be damaged (nude sperm).

### Label-Free Quantitative Proteomics Analysis

#### Protein Extraction and Digestion

Three replicates of each group: swim-up-prepared fresh sperm (control), BM, 0.02 μM MitoQ, 1% glycerol, and Mito-glycerol were prepared for proteomic analysis. The sperm of three semen donors corresponding to each group were pooled to make one replicate. Protein extraction and digestion were performed as described previously (Kohli et al., 2014). Vitrified samples were warmed at 42°C for 15 s and washed twice with PBS by centrifuging at 800 × *g* for 10 min. Similarly, the swim-up-prepared fresh sperm samples were washed. All steps were performed at room temperature. To the pellet of each replicate, 100 μl lysis buffer [8 M urea in 50 mM triethylammonium bicarbonate (TEAB) containing 50 × protease inhibitor cocktail (20 μl in 1 ml lysis buffer; Roche, Munich, Germany)] was added. To rupture the cells physically, the samples were passed through 29-gauge needles numerous times. The lysates were centrifuged for 15 min at 20,000 × *g* to remove cell debris. Proteins in the supernatants were quantified using the bicinchoninic acid (BCA) method (#23225, Pierce, Thermo Fisher Scientific, IL, United States) following the manufacturer’s instructions. Dithiothreitol was added to a final concentration of 5 mM in the sample and the sample was then vortexed and incubated at 25°C for 1 h to reduce the proteins’ disulfide bonds. The alkylation of cysteine was performed by adding chloroacetamide (final concentration, 40 mM) in the sample; the sample was then vortexed and incubated in the dark for 30 min. After incubation, lysyl endopeptidase protease (0.5 μg/μl) was added at an enzyme/substrate ratio of 1:75 and incubated at 25°C for 4 h. The samples were diluted in 50 mM TEAB to achieve a final concentration of urea less than 2 M. After that, trypsin protease (1 μg/μl) was added at an enzyme/substrate ratio of 1:75 and incubated at 25°C overnight. The next day, the samples were acidified by adding formic acid to a final concentration of 1% to stop enzymatic digestion.

#### Purification of Peptides

After isolation of peptides, salts and buffers were removed using reversed-phase (RP) resins, for which the C18 matrix was used to capture hydrophobic peptides. Next, homemade C18 stop-and-go extraction tips (StageTips) were used for the purification of the in-solution digested samples (Rappsilber et al., 2007). The SDB-RP StageTips were packed in 200 μl pipette tips. The equilibration of StageTips was done as follows: the columns were first washed with a 20 μl 100% methanol solution by centrifugation at 2,600 rpm for 1 min to remove contaminants and non-specifically adsorbed compounds. Buffer B (20 μl, 0.1% formic acid in 80% acetonitrile) was added to the tips and centrifuged at 2,600 rpm for 1 min. Thereafter, buffer A (20 μl, 0.1% formic acid in water) was added to the tips twice and centrifuged at 2,600 rpm for 1.5 and 2.0 min, respectively. After equilibration of the StageTips, each sample was loaded onto a StageTip. Before loading, the samples (acidified with formic acid) were centrifuged at full speed for 5 min to discard pellets, if any. The loaded tips were centrifuged at 2,600 rpm for 5 min. The StageTips were washed once with buffer A (30 μl) and twice with buffer B (30 μl) by centrifuging at 2,600 rpm for 3 min to remove non-binding salts. They were then dried with a syringe and kept at 4°C. The peptides were eluted using volatile organics such as acetonitrile (ACN). The final stage of sample preparation was the concentration of the purified peptides and removal of the volatile components using a vacuum concentrator centrifuge.

#### MS/MS Methods

The purified peptides were analyzed using an LTQ Orbitrap Discovery (Thermo Fisher Scientific, Waltham, MA, United States) mass spectrometer as previously described (Rinschen et al., 2010). Briefly, the analysis was carried out using reversed-phase liquid chromatography coupled to nanoflow electrospray tandem mass spectrometry (MS/MS). Separation of the peptide was completed at a flow rate of 300 nl/min over 90 min [5–7% ACN in 5 min, 7–45% ACN in 60 min, 45–50% ACN in 5 min, and 50–97% ACN in 5 min and washing at 100% buffer A (0.1% formic acid in H_2_O) and buffer B (0.1% formic acid in acetonitrile)]. The MS spectra (*m*/*z* = 300–2,000) of the peptides were obtained in Orbitrap at a resolution of 30,000 (*m*/*z* = 445.12003). The mass spectrometer obtained spectra in “data-dependent mode” and automatically shifted between MS and MS/MS acquisition. Signals with unknown charge states were removed from fragmentation. The five most intense ions (charge state: *z* ≥ 2) were isolated and fragmented in the linear ion trap by collision-induced dissociation fragmentation.

### Bioinformatics Analysis

MaxQuant (version 1.6.14.0) was used to analyze the acquired LC-MS/MS raw data. The target-decoy strategy (reversed database) was utilized to search raw data spectra in the human UniProt reference database. The protein, peptide, and site false discovery rate (FDR) was set at 0.01. Label-free quantification (LFQ) analysis was used for proteins identified with one or more peptides. Perseus (Cox and Mann, 2012) software was used to analyze the LFQ intensities for the identified proteins as previously described (Hubner and Mann, 2011). Briefly, raw LFQ intensities were logarithmized and normalized by subtraction of the mean. The protein group had at least two LFQ values that were analyzed. From the dataset, contaminants and proteins resolved by posttranslational modification (PTM) sites were removed. Data imputation was performed based on a normal distribution of the LFQ intensities to replace non-quantified values with low intensities. LFQ quantification was performed for razor and unique peptides. For relative quantification, only proteins that were identified and quantified in at least one biological replicate in each group were used. To identify significant changes between the control and treatment samples among the three biological replicates, a Student’s *t*-test was performed. FDR-based multiple hypothesis testing was used for correction of the *p*-value. The mass spectrometry proteomics data have been deposited to the ProteomeXchange Consortium *via* the PRIDE (Perez-Riverol et al., 2019) partner repository with the dataset identifier PXD025939.

### Statistical Analyses

Statistical analyses of sperm function test data were conducted using IBM SPSS Statistics for Windows, version 21.0 (IBM Corp, Armonk, NY, United States). Numerical data are presented as the mean ± SE. Shapiro–Wilk test was used for normal distribution of data. Levene’s test was used to evaluate the homogeneity of variance. Arcsine transformation of data was performed if the data were not normally distributed. Analysis of variance (ANOVA) was used to compare differences among groups. The least-square mean differences were considered significant when the *p*-value was < 0.05.

## Results

### Tolerance of Spermatozoa Against MitoQ

The tolerable doses (0, 0.02, 0.2, 2, and 20 μM) of MitoQ for human spermatozoa cells were investigated by incubating the spermatozoa with MitoQ for 24 h, and spermatozoa motility was assessed just after addition and after 2, 8, and 24 h. Up to 0.2 μM MitoQ had a positive effect on spermatozoa motility during the incubation period, but at 2 and 20 μM potentially decreased the spermatozoa motility. Therefore, MitoQ concentrations of 0.02 and 0.2 μM were used in subsequent experiments.

### Effect of MitoQ and Glycerol on Sperm Motility and Plasma Membrane Integrity

**FIGURE 1:**
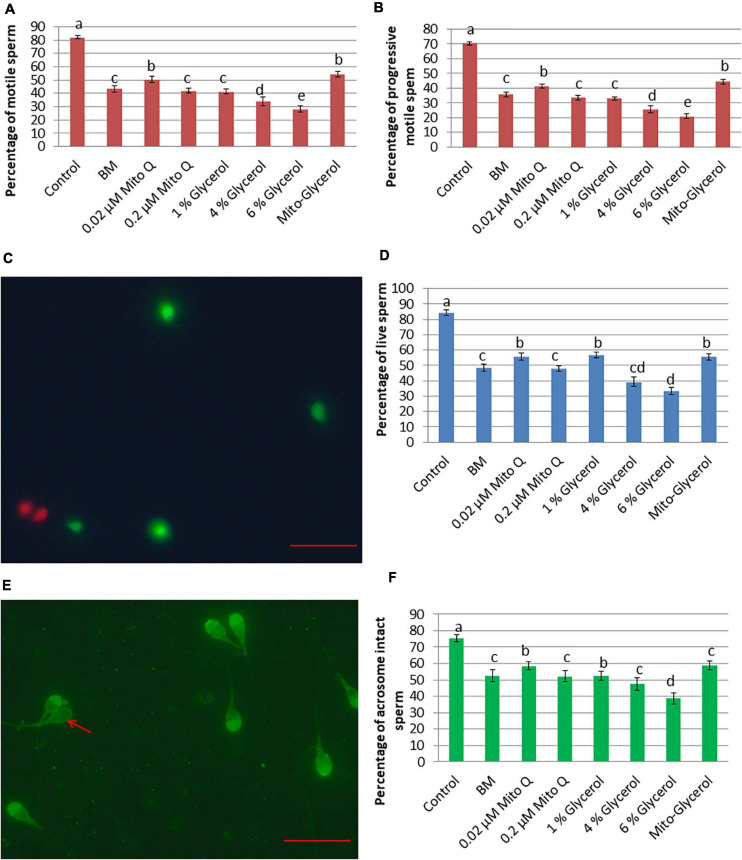
Effect of vitrification on spermatozoa motility, vitality, and acrosomal intactness. **(A)** Total spermatozoa motility. **(B)** Progressive spermatozoa motility. **(C)** SYBR/PI staining. *Green* spermatozoa head indicates live spermatozoa, while *red* spermatozoa head indicates dead spermatozoa. **(D)** Percent live spermatozoa in fresh and vitrified spermatozoa. **(E)** *Pisum sativum* agglutinin lectin–fluorescein isothiocyanate (PSA-FITC) staining. Intact acrosome: bright fluorescence with clear-cut acrosomal cap; damaged acrosome: no fluorescence or only fluorescence of the equatorial segment or irregular acrosomal cap (*red arrow*). **(F)** Percent intact acrosome in fresh and vitrified spermatozoa. *MitoQ*, mitoquinone; *BM*, basal vitrification medium; *Gly*, glycerol; *Mito-Gly*, 0.02 μM MitoQ and 1% glycerol. Values with *different letters* differ significantly (*p* < 0.05). *n* = 20, where *n* is the number of times the experiment was replicated. *Scale bar* = 50 μm.

After spermatozoa vitrification and warming, the total and progressive spermatozoa motility values were higher in the 0.02 μM MitoQ- and Mito-glycerol-supplemented groups than those in BM (Figures 1A,B). After vitrification/warming, the intactness of the plasma membrane of the vitrified spermatozoa was significantly reduced compared to fresh spermatozoa. But when comparing among the vitrified groups, the 0.02 μM MitoQ, 1% glycerol, and Mito-glycerol groups had high percentages of spermatozoa with an intact plasma membrane (live spermatozoa) relative to the basal medium group (Figures 1C,D).

### Effect of MitoQ and Glycerol on Acrosome Membrane Intactness

Cryopreservation causes extensive damage to the acrosome, which might contribute to loss of fertility. Therefore, we estimated the percentage of spermatozoa with an intact acrosome in different groups after vitrification/warming. After vitrification and warming, the 0.02 μM MitoQ and 1% glycerol groups had greater (*p* < 0.05) percentages of intact acrosomes than do the BM and other groups (Figures 1E,F).

### Effect of MitoQ and Glycerol on Mitochondrial Membrane Potential (Δ*Ψ*_*m*_)

**FIGURE 2:**
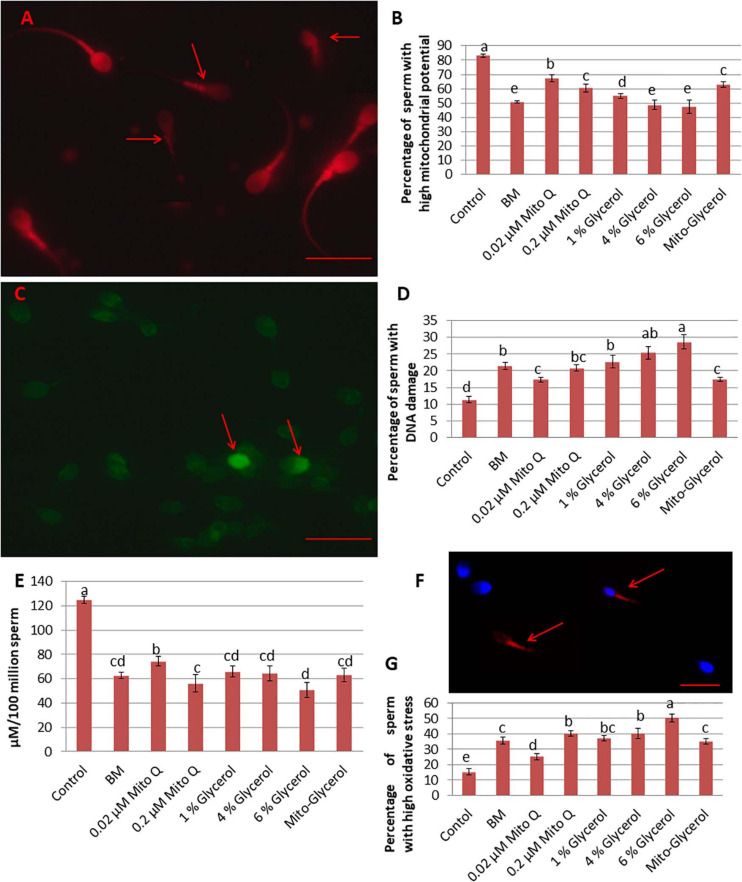
Effect of vitrification on spermatozoa mitochondrial membrane potential, DNA damage, ATP damage, and oxidative stress. **(A)** Tetramethylrhodamine (TMRM) staining. High mitochondrial potential: high fluorescence of the mid-piece region of the spermatozoa; low mitochondrial potential: low fluorescence of the mid-piece region of the spermatozoa (*red arrow*). **(B)** TUNEL staining. *Light green* spermatozoa head: intact DNA; *bright green* spermatozoa head: damaged DNA (*red arrow*). **(C)** Percentage of spermatozoa with high mitochondrial potential in fresh and vitrified spermatozoa. **(D)** Percentage of spermatozoa with damaged DNA in fresh and vitrified spermatozoa. **(E)** ATP production per 100 million spermatozoa by fresh and vitrified spermatozoa. **(F)** CellROX Deep Red staining. Middle piece stained *pale red* or *no color*: low oxidative stress; middle piece stained intense red (*red arrow*): high oxidative stress. **(G)** Percentage of spermatozoa showing high oxidative stress in fresh and vitrified spermatozoa. *MitoQ*, mitoquinone; *BM*, basal vitrification medium; *Gly*, glycerol; *Mito-Gly*, 0.02 μM MitoQ and 1% glycerol. Values with *different letters* differ significantly (*p* < 0.05). *n* = 20, where *n* is the number of times the experiment was replicated. *Scale bar* = 50 μm.

To better evaluate the effect of MitoQ and glycerol on sperm vitrification, the mitochondrial membrane potential of the spermatozoa was estimated as it is an essential component in the process of energy storage during oxidative phosphorylation. In the swim-up-prepared sperm, an average of 83% spermatozoa had a high Δ*Ψ*_*m*_. After vitrification, this was reduced to 50.66% in the basal medium group, but supplementation of 0.02 μM MitoQ significantly improved Δ*Ψ*_*m*_ in the vitrified spermatozoa (Figures 2A,B).

### Effect of MitoQ and Glycerol on DNA Fragmentation

Vitrification and warming led to a significant increase in the percentage of DNA fragmentation. In the swim-up-prepared fresh sperm (control), the proportion of fragmented DNA was about 11%, which increased to 21.4, 17.28, 20.74, 22.65, 25.36, 28.61, and 17.42% in the BM, 0.02 μM MitoQ, 0.2 μM MitoQ, 1% glycerol, 4% glycerol, 6% glycerol, and Mito-glycerol groups, respectively. Thus, the 0.02 μM MitoQ and Mito-glycerol groups protected DNA fragmentation during the process better than the other groups (Figures 2C,D).

### Effect of MitoQ and Glycerol on ATP Production

In the present study, we found that the swim-up-prepared fresh sperm produced an average of 125 μM ATP/million spermatozoa, which reduced significantly after vitrification. The vitrification media supplemented with 0.02 μM MitoQ produced greater ATP than did the basal medium and glycerol groups (Figure 2E).

### Effect of MitoQ and Glycerol on ROS Production

The detection of ROS in the swim-up-prepared fresh sperm and the vitrified sperm of each treatment group is important to evaluate different vitrification media. We found that the Deep Red^®^ fluorescent reagent exhibited an intense fluorogenic signal in 15% of spermatozoa in the fresh spermatozoa suspension. After vitrification, about 35% of spermatozoa showed an intense fluorogenic signal in the basal medium. The supplementation of 0.02 μM MitoQ resulted in only 25% spermatozoa showing an intense fluorogenic signal, but supplementation with a high concentration of MitoQ had no beneficial effect. Similarly, only the 1% glycerol group showed a low percentage of intense fluorogenic spermatozoa cells among the glycerol groups (Figures 2F,G).

### Effect of Vitrification on the Ultrastructure of Spermatozoa

**FIGURE 3:**
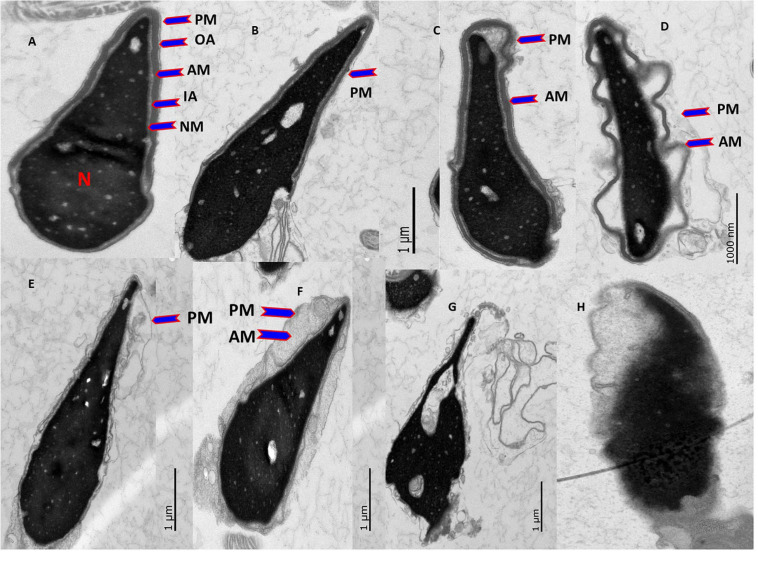
Effect of vitrification on the ultrastructure of spermatozoa heads. TEM images of spermatozoa heads. **(A)** Spermatozoa with undamaged normal heads. *From outward in*: plasma membrane (*PM*), outer acrosomal (*OA*) membrane, acrosomal material (*AM*), inner acrosomal (*IA*) membrane, nuclear membrane (*NM*), and nucleus (*N*). **(B)** Spermatozoa like in **(A)**, except that the PM was damaged. **(C)** Spermatozoa with swollen PM and acrosome. **(D)** Spermatozoa with swollen and wavy PM and acrosome. **(E)** Damaged OA and IA membranes and leaked out AM. **(F)** Damaged OA and IA membranes and condensed AM. **(G)** Detached acrosome and PM, i.e., spermatozoa without a cap. **(H)** Nude spermatozoa: absent IA membrane and damaged NM.

The different types of spermatozoa heads observed under TEM are shown in Table 1. In the swim-up-prepared fresh spermatozoa, type 1 (normal sperm head; Figure 3A) sperm head was the highest, followed by the MitoQ group, then the BM group, and lowest in the glycerol and Mito-glycerol groups. Type 2 sperm head (swollen or broken plasma membrane; Figure 3B) was highest in the MitoQ group and lowest in the glycerol group. Type 3 sperm head (swollen plasma membrane and acrosome; Figures 3C,D) was highest in the Mito-glycerol group and lowest in fresh spermatozoa. Among the vitrified groups, type 4 sperm head (damaged acrosome; Figures 3E,F) was highest in the glycerol group and lowest in the MitoQ group. After vitrification and warming, a few spermatozoa had detached acrosomes. They appeared without a cap (type 5; Figure 3G), and even a few spermatozoa had damaged nuclear material (type 6; Figure 3H). Still, the MitoQ group and fresh spermatozoa had negligible numbers of type 5 and 6 spermatozoa. The overall intact acrosome (types 1, 2, and 3) was greater in the swim-up-prepared spermatozoa, followed by the MitoQ group and lowest in the glycerol group.

**TABLE 1:** Percentage (mean ± SE) of fresh and vitrified spermatozoa with different types of ultrastructural changes in the spermatozoa membrane and acrosome.

	Type 1	Type 2	Type 3	Total of 1–3	Type 4	Type 5	Type 6	Total of 4–6
Fresh	55.78 ± 2.3	12.17 ± 4.4	11.73 ± 1.2	79.68 ± 3.7	20.22 ± 2.5	0.1 ± 0.2	0.0 ± 0	20.32 ± 2.6
BM	12.25 ± 3.2	15.66 ± 3.6	24.52 ± 2.5	52.43 ± 2.3	43.5 ± 2.8	2.27 ± 0.4	1.8 ± 0.2	47.57 ± 3.2
0.02 μM MitoQ	20.1 ± 2.3	35.04 ± 2.4	14.08 ± 1.6	69.22 ± 2.8	30.68 ± 1.5	0.1 ± 0.2	0.0 ± 0	30.78 ± 1.8
1% Glycerol	9.71 ± 4.5	10.47 ± 1.2	25.21 ± 0.9	45.39 ± 2.1	49.66 ± 1.6	2.7 ± 0.3	2.25 ± 0.2	54.61 ± 2.3
Mito-Gly	8.21 ± 2.5	16.66 ± 0.45	30.33 ± 1.3	55.2 ± 3.1	38.25 ± 1.2	3.2 ± 0.3	3.35 ± 0.1	44.8 ± 1.3

*Type 1: spermatozoa with normal heads with an intact plasma membrane (PM), outer acrosomal (OA) membrane, acrosomal material (AM), inner acrosomal (IA) membrane, nuclear membrane (NM), and a nucleus (N). Type 2: Like type 1, except for swollen or damaged PM. Type 3: Like type 2, except for swollen and wavy acrosome. Type 4: Damaged OA and IA membranes and leaked out, dispersed, or condensed AM. Type 5: Detached acrosome and PM and appeared without a cap. Type 6: Damaged NM (nude spermatozoa). *MitoQ*, mitoquinone; *BM*, basal vitrification medium; *Mito-Gly*, 0.02 μM MitoQ and 1% glycerol.*

### Identification of Differentially Expressed Proteins Among the Different Treatment Groups

**FIGURE 4:**
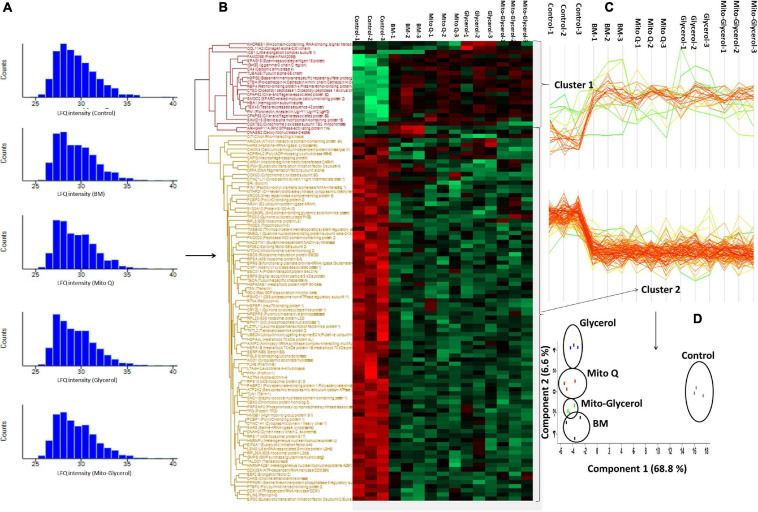
**(A)** Histograms of the label-free quantification (LFQ) intensities of the identified proteins in the control and treatment groups showing a mostly normally distributed pattern. **(B)** Heat map of significant changes in protein levels in the spermatozoa before and after vitrification. The relative abundance level of each protein is shown on a color scale from *green* (lowest) to *red* (highest). **(C)** Profile plots of two distinguished clusters in a heat map (*different colors* of dendrograms) showing distinct behavior from the five states (one control and four treatments). **(D)** Principal component analysis of the differentially expressed proteins showing a well-separated cluster of fresh and vitrified spermatozoa groups. Each *square* represents one sample, and three replicates of each group are enclosed in *circles*.

**FIGURE 5:**
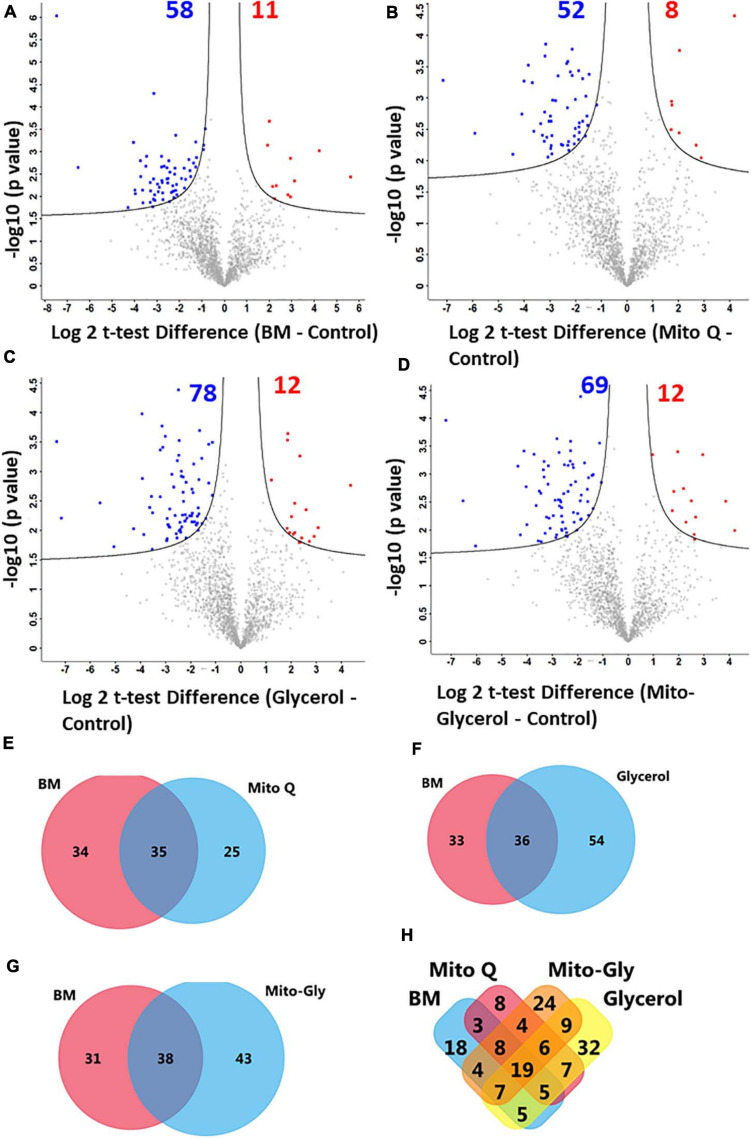
Volcano plots were constructed to identify differentially upregulated and downregulated proteins between vitrified and fresh spermatozoa. Each *point* represents a protein and the *different colors* denote the differential expression pattern: *red* for upregulation, *gray* for flat regulation, and *blue* for downregulation. **(A)** BM vs. fresh. **(B)** MitoQ vs. fresh. **(C)** Glycerol vs. fresh. **(D)** Mito-Gly vs. fresh. *Black solid lines* represent the significance threshold at FDR = 0.01 and *S*_0_ = 0.1. **(E**–**H)** Venn diagrams comparing the differentially expressed proteins among the treatment groups: **(E)** BM vs. MitoQ. **(F)** BM vs. glycerol. **(G)** BM vs. Mito-Gly. **(H)** All treatment groups. *MitoQ*, mitoquinone; *BM*, basal vitrification medium; *Mito-Gly*, 0.02 μM MitoQ and 1% glycerol.

Label-free proteomic quantification was conducted to recognize changes in the sperm proteome following vitrification to identify functional proteins susceptible to the vitrification process in the different treatment groups. A total of 1,759 proteins were detected in human spermatozoa (Supplementary Table 1). To verify whether the identified proteins in the control and treatment groups were distributed normally, we plotted histograms of the LFQ intensities of the identified proteins. We found that the histograms of all groups are mostly normally distributed, indicating the robustness of the MS/MS analysis and sample homogeneity (Figure 4A). Next, multi-scatter plots with Pearson’s correlations among three replicates of the control and vitrified spermatozoa were analyzed (Supplementary Figure 1). The *R*^2^ values ranged from 0.538 to 0.783 among three replicates of the control and vitrified spermatozoa. Among the vitrified groups, *R*^2^ values ranging from 0.798 to 0.917 indicated a clear-cut difference between each replicate of fresh and vitrified spermatozoa. However, the multi-scatter plot matrix of the identified proteins failed to distinguish among vitrified spermatozoa. To visualize how the levels of these differentially altered proteins changed in each sample, we performed hierarchal clustering of the logarithmic protein levels before and after vitrification (Figure 4B). The profile plots of two distinguished clusters in a heat map (different colors of dendrograms) showed a distinct behavior for fresh and vitrified spermatozoa (Figure 4C). Principal component analysis (PCA) revealed that principal components 1 and 2 represent 75.00% of the total variance of the dataset (68.4% and 6.6%, respectively). This means that the first principal component alone explained a very large component of the data. The second principal component explained less than the first major component, but more than all the other components. The well-separated clustering of the control and vitrified spermatozoa groups indicated that swim-up-prepared fresh sperm samples are distantly separated from the treatment samples. Among the vitrified spermatozoa, all the clusters were well separated, but the variance is lower compared to the control. Furthermore, the PCA plots revealed that the replicates for each of the experimental groups were closely clustered, suggesting high consistency between replicates of each group (Figure 4D). Finally, to identify altered proteins after vitrification, volcano plots were constructed. A total of 118 proteins were in low abundance and 21 proteins were in high abundance after vitrification (Supplementary Tables 2, 3). For the relative quantification of proteins, the data were log_2_-transformed. This means that a unit change of 1 corresponds to a twofold difference. The log2 difference between two samples was plotted on the *x*-axis, while the *p*-value of the *t*-test performed from triplicate experiments between the vitrified and the control spermatozoa was plotted on the *y*-axis. For better visualization, the *p*-values were log_10_-transformed and multiplied by –1. This means that a value of 1 corresponds to a *p*-value of 0.1 and a value of 2 corresponds to a *p*-value of 0.01. The larger the significant difference, the smaller the *p*-value and the higher the –log_10_(*p*) value. The threshold values to filter statistically significant changes in Perseus, an FDR of 0.01 and an *S*_0_ parameter of 1, were preferred. We considered *S*_0_ = 0.2 for the volcano plot because in the volcano plot, at *S*_0_ = 0, only the *p*-value matters. At the same time, at nonzero *S*_0_, the difference of the means plays a role in identifying the differentially expressed proteins. Spermatozoa vitrification in the basal medium resulted in 69 altered proteins compared to the control. Out of 69 proteins, 58 proteins were in low abundance and 11 proteins were highly abundant (Figure 5A) compared to the control group. Spermatozoa vitrified in basal medium supplemented with 0.02 μM MitoQ resulted in 60 altered proteins compared to the control group. Of the 60 proteins, 52 proteins were found in low abundance and eight proteins were highly abundant (Figure 5B). Spermatozoa vitrification in basal medium supplemented with 1% glycerol resulted in 90 altered proteins compared to the control group. Of these 90 proteins, 78 proteins were found in low abundance and 12 proteins were highly abundant (Figure 5C). Sperm vitrification in basal medium supplemented with 0.02 μM MitoQ and 1% glycerol resulted in 81 differentially altered proteins compared to the control group. Of these 81 proteins, 69 proteins were found in low abundance and 12 proteins were highly abundant (Figure 5D). From these results, it appears that supplementation of MitoQ protects spermatozoa proteins from alterations, while supplementing glycerol results in more alterations of spermatozoa protein during the vitrification process. We performed a Venn diagram analysis to determine which altered proteins are common among the different treatment groups. Twenty-five, 54, and 43 differentially expressed proteins in the 0.02 μM MitoQ, 1% glycerol, and Mito-glycerol groups, respectively, did not overlap with the basal medium group, while 35, 36, and 38 differentially expressed proteins did overlap with the basal medium group (Figures 5E–G). The Venn diagrams show that the lowest and highest non-overlapped altered proteins were found in the MitoQ and glycerol groups, respectively. Among all the treatment groups, 19 differentially expressed proteins were common in all four treatment groups, indicating that these proteins are susceptible to alterations during vitrification irrespective of the vitrification media used in the study (Figure 5H and Supplementary Table 4). However, 18, 8, 32, and 24 proteins (Figure 5H and Supplementary Tables 5–8) were altered in the basal medium, 0.02 μM MitoQ, 1% glycerol, and Mito-glycerol groups, respectively, indicating that these proteins are exclusively susceptible to cryo-damage during the vitrification process to the particular groups. It is interesting to note that only eight differentially altered proteins were exclusively susceptible in the MitoQ group, indicating that spermatozoa proteins are less susceptible to cryo-damage during the vitrification process. On the other hand, higher numbers of proteins were more susceptible to alterations in the glycerol group during the vitrification process.

### Classification of the Differentially Expressed Proteins Based on Their Cellular Location, Molecular Function, and Role in Biological Processes

To better understand the functional relevance of the identified proteins, we used Funrich software (Pathan et al., 2015) to classify the differentially altered proteins involved in major molecular functions and biological processes and analyze their cellular localization. Cellular component analysis revealed that proteins of presumed cellular location damaged during vitrification are minimum in MitoQ and maximum in the glycerol-supplemented group, particularly for the cytoplasm, nucleus, mitochondrion, acrosome, F-actin capping protein complex, and dynein complex cellular components (Supplementary Table 9). Molecular function analysis revealed that, in proteins belonging to transport activity, hydrolase activity, chaperone activity, oxidoreductase activity, RNA binding, protein serine/threonine kinase activity, and heat shock protein activity, damage/denaturation was minimal in the MitoQ-supplemented groups (Supplementary Table 10). Similarly, biological process analysis reveals that proteins that belong to metabolism, energy pathways, protein metabolism, signal transduction, cell communication, and transport process were minimally affected during spermatozoa vitrification by supplementing MitoQ in basal medium (Supplementary Table 11).

### Major Classes of Sperm Proteins Affected by Vitrification

The actin and actin-associated proteins influence a wide range of functions in the spermatozoa cell, including shape, movement, and interactions with extracellular matrices. We identified several actin and actin-binding proteins in the spermatozoa, some of which are differentially altered in particular treatment groups (Supplementary Table 12). Tubulin is the protein that polymerizes into long chains or filaments and forms microtubules surrounding the axoneme of the spermatozoa tail. Several tubulins were identified, and none of them was affected during vitrification (Supplementary Table 13). The ubiquitin–proteasome system has a central role in signal transduction, protein trafficking, apoptosis, and fertilization of the spermatozoa. Several ubiquitinating and deubiquitinating enzymes and subunits of proteasome were identified in the human spermatozoa, some of which were altered during vitrification (Supplementary Table 14). Phosphorylation/dephosphorylation is a dynamic process involving protein kinases and protein phosphatases. Several kinases and phosphatases have been identified in the spermatozoa, some of which are altered during vitrification (Supplementary Table 15). Several heat shock proteins were also identified in human spermatozoa and were not affected during vitrification, except the DnaJ homolog (Supplementary Table 16). Several candidate proteins involved in spermatozoa–egg interactions were also identified in the spermatozoa, but not damaged during spermatozoa vitrification (Supplementary Table 17).

## Discussion

Here, we describe MitoQ alleviated while glycerol increased oxidative stress during the vitrification process. To protect the spermatozoa from ice crystals during the slow-freezing method of sperm cryopreservation, glycerol, a permeable cryoprotectant, is added in the freezing medium. Glycerol is toxic for spermatozoa, which induces massive osmotic stress results in dehydration and alterations in the sperm head volume (Barbas and Mascarenhas, 2009). Simultaneously, glycerol causes the rearrangement of membrane proteins and lipids of the spermatozoa and excessive ROS generation, resulting in the disruption of mitochondrial membrane potential, alteration of membrane permeability, and damage of sperm surface proteins (Liu and Foote, 1998). During the slow-freezing method of cryopreservation, the benefits of the addition of glycerol in the freezing medium outweighed its side effects. In the case of sperm kinetic vitrification, there was no issue of ice crystal formation because of the rapid cooling rate. Therefore, the addition of glycerol in the vitrification medium induces excessive oxidative stress. The increased oxidative stress results in redox-dependent protein modifications (Morielli and O’Flaherty, 2015). Protein modifications during vitrification impacted motility, membrane integrity, ATP production, and DNA integrity, etc. We discussed in detail how sperm vitrification causes qualitative and quantitative changes in sperm and that MitoQ ameliorates the changes by reducing the oxidative stress during the vitrification process.

### MitoQ Alleviated Oxidative Stress During the Vitrification Process

To our knowledge, this is the first study demonstrating that MitoQ protects the mitochondria from oxidative stress during the sperm vitrification process. In the present study, we have demonstrated that 0.02 μM MitoQ improved the spermatozoa quality after vitrification. Still, at higher concentrations, favorable effects were not observed. To counterbalance the excess production of ROS during cryopreservation, the antioxidants present inside the spermatozoa mitochondria are not sufficient. Therefore, supplementation of antioxidants from outside has been a common method, but only an insufficient quantity of the antioxidants crosses the inner mitochondrial membrane. The findings indicate that the active component of MitoQ (coenzyme Q10 or ubiquinone) reaches a sufficient concentration to the inner mitochondrial membrane to combat the excess production of ROS during the vitrification process. After detoxifying the excess ROS generated during vitrification, the quinones of MitoQ are restored by the respiratory chain, allowing its antioxidant activity to be recycled (Kelso et al., 2001). Thus, the supplemented MitoQ continuously supports ATP production to maintain spermatozoa motility for a longer period and neutralizes excess ROS, resulting in less damage to the plasma membrane, acrosome, DNA, and spermatozoa proteins during the vitrification process. Spermatozoa proteins are sensitive to high levels of ROS and suffer modifications that impact spermatozoa motility and fertilization (O’Flaherty and Matsushita-Fournier, 2017). The electron microscopy and proteomic results would support our hypothesis that MitoQ protects the spermatozoa during the vitrification and warming processes.

On the other hand, we found that if MitoQ is supplemented in a large dose (0.2 μM), it is toxic to the spermatozoa. Similar to our findings, another study also suggested that a slight increase (even at micromolar concentrations) of MitoQ can interrupt membrane integrity, leading to the malfunction of mitochondrial activity (Cochemé et al., 2007). Since MitoQ accumulates into the mitochondria at very high concentrations, the conjugate decyl-triphenylphosphonium (dTPP) concentration also increases. It has been demonstrated that dTPP compounds inhibited oxidative phosphorylation (Reily et al., 2013) and mitochondrial potential (Trnka et al., 2015). This might be the reason for the lower production of ATP, reduction of mitochondrial potential, and the increased ROS production in the spermatozoa vitrified in the vitrification medium containing a high concentration of MitoQ. Therefore, it is necessary to determine the correct dose of MitoQ for spermatozoa of different species due to great variability in the spermatozoa membrane composition of cholesterol and saturated and unsaturated phospholipids. Similar to our study, other studies have also used other mitochondria-targeted antioxidants for the cryopreservation of human (Lu et al., 2018; Zhang et al., 2019), boar (Cho et al., 2020), and rooster (Masoudi et al., 2020) spermatozoa with the slow-freezing method and found better total motility, progressive motility, membrane integrity, and antioxidant status compared to the control groups.

### Transmission Electron Microscopy Demonstrated That MitoQ Protects Ultrastructural Changes During Vitrification

Spermatozoa must have intact acrosomes to penetrate and fertilize the oocyte (Buffone et al., 2009). The acrosome integrity of spermatozoa is highly susceptible to elevated ROS (Mostafa et al., 2016) because of the specialized structure of the acrosome, consisting of membranes and proteins, which renders it sensitive to high levels of ROS (HuangJr., and Yanagimachi, 1985). It can be speculated that lipid peroxidation of spermatozoa membranes and the oxidation of acrosomal proteins during vitrification adversely affect the acrosomal structure and function. The addition of MitoQ in the vitrification medium sufficiently reduced the oxidative stress of spermatozoa during the vitrification and warming processes, resulting in less damage to the spermatozoa plasma membrane, acrosomal proteins, and, finally, to the ultrastructure of the acrosome. On the other hand, the addition of glycerol in the vitrification medium increased the oxidative stress of the spermatozoa, resulting in significant alterations in the ultrastructural morphology of the acrosomes of vitrified spermatozoa. Mahadevan and Trounson (1984) reported that types 1, 2, and 3 were positively correlated with spermatozoa fertility, whereas types 4, 5, and 6 were negatively correlated. After vitrification, a high proportion of type 1, 2, and 3 spermatozoa heads was found in the MitoQ group, indicating that MitoQ gave better protection of spermatozoa heads during the vitrification process among all treated groups. Jones and Stewart (1979) reported that acrosomal swelling is a preliminary stage of acrosomal reaction, and it does not affect the fertilizing capacity of the spermatozoa. High percentages of type 4, 5, and 6 spermatozoa heads were found in the glycerol group, indicating that glycerol is detrimental for spermatozoa vitrification. It is interesting to note that Jones and Stewart (1979) and Mahadevan and Trounson (1984) found 9.8% and 8.8% of frozen/thawed bull and human spermatozoa, respectively, as normal using a similar classification system. These findings indicate that the vitrification process is a considerably less damaging process than the slow-freezing method (20.1% vs. 9.8% vs. 8.8%). Also, it is clear that supplementing the basal vitrification medium with MitoQ at 0.02 μM results in a better ultrastructure of the vitrified spermatozoa than that in other groups.

### Label-Free Quantitative Proteomics Revealed Alterations in Key Proteins During Spermatozoa Vitrification

It is clear from TEM that supplementing the basal vitrification medium with 0.02 μM MitoQ results in better semen quality of the vitrified spermatozoa. However, these laboratory tests and TEM fail to explain the alterations in the proteins at the subcellular level of spermatozoa during spermatozoa vitrification. Although several studies reported successful vitrification of human spermatozoa, no report has addressed the changes in the proteomics profiles of fresh and vitrified spermatozoa. Therefore, we used label-free quantitative proteomics analysis to reveal the key proteins altered during the vitrification process. Earlier, proteomics approaches were used to study the alterations in proteins after cryopreservation of human sperm using slow-freezing methods (Wang et al., 2014; Bogle et al., 2017; Fu et al., 2019; Li et al., 2019). Wang et.al (2014) found only 27 proteins that differed in abundance between fresh and cryopreserved sperm due to using low-sensitivity two-dimensional polyacrylamide gel electrophoresis (2-DE). Bogle et al. (2017) identified proteomic changes at every stage of the cryopreservation process using tandem mass tag technology, but they did not compare fresh and cryopreserved sperm. Fu et al. (2019) revealed through the isobaric tags for relative and absolute quantitation (iTRAQ) approach that 432 proteins were differentially identified. Of these, 115 proteins increased and 317 proteins decreased after cryopreservation of sperm. Li et al. (2019) identified proteomic changes during human sperm cryopreservation using data-independent acquisition mass spectrometry. A total of 174 significantly differential proteins were identified between fresh and cryopreserved sperm. Of these, 98 proteins decreased and 76 proteins increased in the cryopreservation group. We identified alterations in proteins during human sperm cryopreservation by using a label-free quantitative approach, a more accurate technique useful for quantifying differences of low abundance proteins (Dowle et al., 2016), and 60–90 differential proteins were identified between fresh and cryopreserved sperm among the different treatment groups. From the study, as a whole, it appears that a small number of proteins are altered during sperm vitrification compared to using slow-freezing methods. The exact reason for the differential abundance of proteins after cryopreservation is still unknown. Mature spermatozoa are said to be both transcriptionally and translationally silent. Therefore, the alterations in the sperm proteome may be due to posttranslational modifications, interaction of the spermatozoa with the freezing medium before cryopreservation, membrane damage during cryopreservation resulting in the efflux of cytoplasmic proteins, or a damaged sperm membrane facilitating more protein extraction during sample preparation for MS. Whatever the reason for the differential abundance of proteins, it is certain that the alterations of these proteins affect sperm functionality and the fertilizing ability of cryopreserved spermatozoa. Several studies have identified a large catalog of integral sperm proteins, but we will focus on those that have greater significance to sperm function and may have an effect on fertility outcomes.

### Effect of Vitrification on Actin and Actin-Binding Proteins

Actin is found in the head and the tail of the spermatozoa and is involved in capacitation, acrosomal exocytosis, and spermatozoa motility. We identified actin, cytoplasmic actin 2, alpha-actinin-1, and alpha-actinin-4, but none of these were affected by the vitrification process, but conventional slow-freezing processes resulted in the loss/damage of the cytoskeleton actin in different species (Felipe-Pérez et al., 2012; Naresh, 2016). The polymerization of globular (G)-actin to form filamentous (F)-actin is controlled by several actin-binding proteins (Breitbart et al., 2005). A plethora of actin-binding proteins is known to control actin assembly and disassembly. We detected actin-like proteins, actin-related protein (Arp) 2/3 complex, actin-related protein, F-actin-capping protein subunit, dynactin subunit, spectrin, calpain, attractin, vinculin, talin, dynein, tropomyosin, tropomodulin-3, vasodilator-stimulated phosphoprotein, thymosin beta-4, and profilin. The F-actin-capping protein (CAPZ) family is a major cytoskeletal protein. The capping proteins are involved in assembly and disassembly during capacitation in the outer acrosomal membrane (Dvoráková et al., 2005). Bogle et al. (2017) found that the F-actin-capping protein subunit alpha-3 is altered in frozen-thawed spermatozoa compared to fresh spermatozoa. On the other hand, Yoon et al. (2016b) reported a higher expression of the F-actin-capping protein subunit beta (CAPZB) in epididymal spermatozoa after the conventional slow-freezing method. We detected CAPZA1, CAPZA2, CAPZA3, and CAPZB, but they remained unaffected during the vitrification process. Spectrin acts as a spring and alters the elasticity of actin (Discher and Carl, 2001). It is suggested that it provides elasticity to the flagella during spermatozoa hyperactivation (Gervasi et al., 2018). Spectrin beta chain, non-erythrocytic 1 was low in spermatozoa vitrified in the BM and glycerol groups, but was unaffected in the MitoQ group. Among the actin-like proteins (2, 3, 7A, 7B, and 9), the actin-like protein 7B was significantly altered when the spermatozoa were vitrified in the basal vitrification medium. Dyneins are cytoskeletal motor proteins that move along microtubules in cells. They convert the chemical energy stored in ATP to mechanical work. We detected six cytoplasmic and 10 axonemal dyneins in human spermatozoa. Among these dynein proteins, axonemal dynein heavy chain 2 was affected by the vitrification in all groups. In contrast, cytoplasmic dynein 1, heavy chain 1, and intermediate chain 1 were not affected by the glycerol group. Li et al. (2019) and Bogle et al. (2017) also reported alterations of axonemal and cytoplasmic dyneins in spermatozoa cryopreserved by the slow-freezing method, indicating that dyneins are susceptible to cryopreservation irrespective of the cryopreservation method. Vinculin and talin are cytoskeletal proteins associated with focal adhesion between cells (Hall et al., 2011). We found that vinculin was not affected during the vitrification process. Talin-1, found in the acrosomal region of the spermatozoa and is involved in capacitation (Brown and Turner, 2004), was altered in all vitrified spermatozoa irrespective of groups. Tropomyosin is found in the principal piece of the tail (Yagi and Paranko, 1992) and was altered in vitrified spermatozoa in the glycerol group. Tropomodulin-3, a protein that regulates the length of actin filaments (Rao et al., 2014), was found to be altered in all vitrified spermatozoa, except in the BM group. Thymosin beta-4 and profilin regulate the rate and extent of actin polymerization in cells (Goldschmidt-Clermont et al., 1992). Thymosin beta-4 was altered in spermatozoa of the glycerol and Mito-glycerol groups, but profilin-1 and profilin-2 were unaffected during spermatozoa vitrification. Thus, actin proteins were unaffected during spermatozoa vitrification. Still, a few actin-binding proteins were altered in vitrified spermatozoa, and supplementation of MitoQ, but not glycerol, appears to prevent alterations of the proteins during the vitrification process.

### Effect of Vitrification on Tubulin and Tubulin-Associated Proteins

The axoneme comprises two central microtubules surrounded by nine microtubule doublets. Microtubules are composed of α-tubulin and β-tubulin. During traditional slow-freezing spermatozoa cryopreservation, high abundance of α- and β-tubulins and low abundance of tubulin polymerization promoting proteins (TPPPs) have been reported (Desrosiers et al., 2006; Gholami et al., 2018; Dariush et al., 2019). Gholami et al. (2018) reported more abundance of tubulin α-1B chain, tubulin α-3C/D chain, tubulin β-2C chain, and tubulin β-2B chain and low abundance of TPPP2 after human spermatozoa cryopreservation. Desrosiers et al. (2006) proposed that the increase in tubulin expression might be due to the freezing and thawing processes that weaken the tubulin-containing structures in the spermatozoa cell. Therefore, the protein is more susceptible to detergent extraction. However, Gholami et al. (2018) assumed that the increased expressions of α- and β-tubulins might be due to microtubule depolymerization during the cryopreservation process. Among the tubulin proteins, we detected tubulin α-1A chain, tubulin α-1B chain, tubulin α-1C chain, tubulin α-3E chain, tubulin α-4A chain, tubulin beta chain, tubulin β-2A chain, tubulin β-4B chain, tubulin β-4A chain, tubulin β-8 chain, and TPPP2. Interestingly, in this study, the tubulin proteins and TPPP2 were unaffected during the vitrification process.

### Effect of Vitrification on Outer Dense Fibers and Vimentin

The outer dense fibers (ODFs) are tail-specific cytoskeletal structures of the spermatozoa (Fawcett, 1975). They comprise nine fibers surrounding the axoneme from the mid-piece to the principal piece of the spermatozoa tail. The expression levels of the ODF major components (ODF1, ODF2, ODF3, and ODF4) were generally low in abundance in asthenozoospermatic men (Zhao et al., 2018). Thus, ODFs are involved in maintaining spermatozoa structure and movement (Yamaguchi et al., 2014). The absence of ODFs results in non-functional tails and affects spermatozoa motility (Yuan, 2014). ODF2 abundance was reported to be decreased after conventional slow freezing (Yoon et al., 2016b). In the present study, we detected ODF1, ODF2, ODF3, and ODF3B, and none of these ODFs were affected during the vitrification process. In addition, we detected tubulin-associated proteins in the spermatozoa, such as tubulin-tyrosine ligase-like protein 12, tubulin-folding cofactor B, tubulin polymerization-promoting protein family member 2, and tubulin-specific chaperone A and D. Of these, only tubulin-specific chaperone A was found to be in low abundance in vitrified spermatozoa. Vimentin has been detected in the head of human spermatozoa (Virtanen et al., 1984). Wang et al. (2014) suggested that the damaged acrosome in frozen–thawed spermatozoa is due to the degradation of the cytoskeletal components, such as vimentin. Still, in the present study, vimentin was found to be unaffected during the vitrification process.

### Effect of Vitrification on Spermatozoa Ubiquitination and Deubiquitination

Ubiquitination has a key role in protein degradation, while deubiquitination has the opposite role. In ubiquitination, with the help of ubiquitin-activating enzyme (E1), ubiquitin-conjugating enzyme (E2), and ubiquitin ligase (E3), ubiquitin molecules are attached to protein substrates for protein degradation. The mitochondrial DNA is removed by ubiquitination during the early stages of embryogenesis (Sutovsky et al., 1999). We detected many ubiquitinating enzymes (ubiquitin-conjugating enzyme E2 variant 1, ubiquitin-conjugating enzyme E2 R1, conjugating enzyme E2 N-like, ubiquitin-conjugating enzyme E2 N, ubiquitin-conjugating enzyme E2 variant 1, ubiquitin-conjugating enzyme E2 R1, ubiquitin-conjugating enzyme E2 N, and ubiquitin-conjugating enzyme E2 D2). Interestingly, all the identified ubiquitinating enzymes were unaffected, except for ubiquitin-conjugating enzyme E2 R1 and ubiquitin-conjugating enzyme E2 R1 that were in low abundance in the Mito-glycerol group. Protein deubiquitination is much less well understood than protein ubiquitination. At present, the physiological roles of deubiquitinating enzymes in spermatozoa are little known. However, we detected several deubiquitinating enzymes that were not affected during the vitrification process.

### Effect of Vitrification on Phosphorylation and Dephosphorylation

Spermatozoa rely on protein phosphorylation/dephosphorylation for physiological functions, like motility, capacitation, and fertilization. Phosphorylation and dephosphorylation are dynamic processes involving protein kinases and protein phosphatases. The serine, threonine, or tyrosine of proteins are phosphorylated by protein kinases and modify protein functions. The enzymes can increase or decrease the activity of proteins and either stabilize or mark proteins for destruction. There are two main types of protein kinases: serine/threonine kinases and tyrosine kinases. The serine/threonine kinases were not affected during the vitrification process. However, calcium/calmodulin-dependent protein kinase type IV was altered in the Mito-glycerol group’s vitrified spermatozoa. Adenylate kinase activity was detected in the flagella of ejaculated bovine spermatozoa and can generate sufficient ATP to produce normal motility (Schoff et al., 1989). We identified mitochondrial adenylate kinase 2, adenylate kinase 7, adenylate kinase 8, and adenylate kinase isoenzyme 1 in human spermatozoa. Of these, adenylate kinase 2 was found to be in low abundance in the MitoQ group. In the spermatozoa, A-kinase anchoring proteins (AKAPs) restricted on the fibrous sheath are prominent tyrosine-phosphorylated proteins during capacitation (Mandal et al., 1999). We detected AKAP3, AKAP4, and AKAP14, but they were not affected by the vitrification process. Therefore, from the study, it appears that the phosphorylation process is little affected during spermatozoa vitrification. On the other hand, it has been reported that several kinases were altered during spermatozoa cryopreservation with the slow-freezing method (Bogle et al., 2017). Serine/threonine phosphatases remove the phosphate group from proteins. Phosphatase enzymes are essential to many biological functions. In the spermatozoa, the serine/threonine-protein phosphatase PP1-gamma catalytic subunit 2 (PPP1CC2) has been identified in spermatozoa tail and posterior and in equatorial regions of the spermatozoa, suggesting a role in spermatozoa motility and acrosome reaction (Huang et al., 2002). A higher activity of PPP1CC2 was found in immotile spermatozoa than in motile spermatozoa (Huang et al., 2002). We detected several serine/threonine-protein phosphatases (Supplementary Table 13). Only two (serine/threonine-protein phosphatase 6 regulatory subunit 1 and subunit 2) were affected during the vitrification process.

### Effect of Vitrification on Heat Shock Proteins of Spermatozoa

A group of proteins called heat shock proteins (HSPs) protect cells during injury and oxidative stress (Beere and Green, 2001). Heat shock proteins comprise a group of unrelated proteins that are classified into the HSP100 (HSPH), HSP90 (HSPC), HSP70 (HSPA), HSP60 (HSPD), and HSP27 (HSPB) families. On the spermatozoa surface, HSP70 proteins are more abundant (Miller et al., 1992; Boulanger et al., 1995). Many reports indicate that HSPs are involved in spermatozoa development and post-spermatogenesis (Neuer et al., 2000; Amaral et al., 2014; Li et al., 2014). HSPA2 regulates the human spermatozoa–egg interaction (Nixon et al., 2015). The HSP70 expression level decreased in spermatozoa cryopreserved by conventional slow freezing (Zhang et al., 2015). It has also been reported that the abundance of HSP90, which plays a direct role in the motion characteristics of spermatozoa, decreased after spermatozoa cryopreservation with the slow-freezing method (Huang et al., 2009). Bogle et al. (2017) found that the 70-kDa heat shock protein 4L (HSPA4L) level decreased when the spermatozoa were equilibrated in semen media containing cryoprotectants. The protein has also been reported to be in low abundance in low-motile spermatozoa (Amaral et al., 2014), but it was not affected during the vitrification process in our study. DNAJ (HSP40, DNAJA1), a chaperone protein that prevents protein misfolding in a cell, was unaffected during vitrification of the MitoQ and glycerol groups. In addition to DNAJA1, we identified 10 more DNAJ that were unaffected during the vitrification process of any groups in the study (Supplementary Table 14), indicating that HSPs were not susceptible to damage during the vitrification process, unlike in the slow-freezing method.

### Effect of Spermatozoa Vitrification on Functional Spermatozoa Proteins Responsible for Participation in Spermatozoa–Oocyte Interactions

In 2005, Masaru Okabe’s group identified a protein and showed that spermatozoa lacking this protein could not fuse with female gametes (Inoue et al., 2005). The protein was called “Izumo,” after a Japanese marriage shrine. The IZUMO proteins consist of four family proteins (IZUMO1 to IZUMO4) (Ellerman et al., 2009). Yoon et al. (2016a) reported that the expression of IZUMO4 significantly increased after the addition of cryoprotectants in the semen extender due to an increased acrosome reaction pattern. In the present study, we detected IZUMO1, IZUMO2, and IZUMO4, and interestingly, the most important reported proteins responsible for fertilization were unaffected during the vitrification process, unlike with the slow-freezing method. Recently, GLIPR1-like protein 1 (GLIPRL1L1) was identified in the spermatozoa, and loss of this protein results in failure of IZUMO1 relocalization to fuse with eggs (Gaikwad et al., 2019). We also identified GLIPRL1L1 in human spermatozoa and found that the protein was unaffected during the vitrification process. A widely discussed model in mammalian fertilization has been the binding of a spermatozoa ADAM (a disintegrin and metalloprotease) to an egg integrin as a required step for spermatozoa–egg membrane fusion (He et al., 2003). ADAM1, ADAM7, ADAM9, ADAM10, ADAM32, and ADAMTS1 were identified and found to be unaffected during the vitrification process of any treatments and control groups. Calmegin is required to dimerize ADAM1A/ADAM2 and for the maturation of ADAM3 (Ikawa et al., 2011). The knockout mice for calmegin were sterile, but spermatogenesis was normal (Ikawa et al., 2011). Calmegin and calreticulin were found to be unaltered during the vitrification process. Human Tektin-2 was identified in the principal piece of the spermatozoa (Tanaka et al., 2004) and plays a critical role in the formation and development of the flagella of the spermatozoa (Setter et al., 2006). Wang et al. (2014) reported that the low abundance of Tektin-1 might be responsible for the reduced motility in frozen–thawed spermatozoa. Alshawa et al. (2019) also demonstrated the decreased expression of Tektin-2 in cryopreserved spermatozoa. During the vitrification process, Tektin-1, Tektin-2, Tektin-3, Tektin-4, and Tektin-5 were unaffected irrespective of the treatment groups, indicating that the spermatozoa vitrification method is more suitable than the traditional slow-freezing method. During the slow-freezing process, the expressions of the zona pellucida-binding proteins 1 and 2 were decreased (Bogle et al., 2017), but these were unaffected during the vitrification process. The expression of superoxide dismutase 1 (SOD1) was increased in spermatozoa cryopreserved with the slow-freezing method (Chen et al., 2014; Yoon et al., 2016b), but the expressions of SOD1 and SOD2 were unaltered during the vitrification process in the present study. The spermatozoa acrosome-associated (SPACA) family proteins are a less characterized group of proteins found in the acrosome. SPACA3 and SPACA4 are involved in binding the spermatozoa to the egg plasma membrane (Shetty et al., 2003; Herrero et al., 2005). Bogle et al. (2017) found that the abundance of SPACA3 decreased in the spermatozoa after the conventional cryopreservation method. During the vitrification process, SPACA1, SPACA3, SPACA4, and SPACA5 were not cryo-damaged, unlike in slow-freezing methods. Succinyl-CoA:3-ketoacid CoA transferase (OXCT1) enzyme deficiency in the spermatozoa results in consequential compromised glycolysis (Yuan, 2014). The OXCT1 abundance decreased during the conventional slow-freezing method of spermatozoa cryopreservation (Wang et al., 2014), but was not affected during the vitrification process.

### High Abundance of Proteins After Vitrification

The finding of a set of proteins being more abundant in cryopreserved vs. fresh sperm samples creates confusion. There is no clear-cut evidence of the more abundance of some proteins after the cryopreservation process. It is widely accepted that there is no *de novo* protein synthesis in mature sperm. However, some recent literature indicate that mature spermatozoa are capable of using mitochondrial RNA transcripts for protein translation during the final maturation steps before fertilization (Gur and Breitbart, 2006; Zhu et al., 2019). Another possible mechanism might be posttranslational modifications during the cryopreservation process. We cannot overlook systematic bias in the comparative proteomics, i.e., the incubation of samples in the vitrification/cryopreservation medium, the vitrification/freezing, or the combination of both renders certain proteins more accessible to mass spectrometric quantification. The issue of the artifactual increase of the abundance of proteins needs to be investigated. Physiologically, the mammalian acrosome contains several hydrolytic enzymes, and their abundance increases before fertilization due to posttranslational modifications (Tulsiani et al., 1998). Like our studies, the increased abundance of some proteins in the cryopreserved spermatozoa has been reported in many studies (Wang et al., 2014; Bogle et al., 2017; Li et al., 2019). Wang et al. (2014) found that some proteins are higher in the freeze–thawed group due to tyrosine phosphorylation than those in the fresh group due to capacitation-like changes during cryopreservation. In our study, we found that most of those proteins highly abundant in vitrified spermatozoa are reported to localize in the acrosomal and equatorial regions of the spermatozoa and are found to be involved in capacitation, acrosome reaction, spermatozoa–egg fusion, and penetration. During cryopreservation, the number of ROS sharply increases, which activates protein tyrosine phosphorylation (Aitken et al., 1995; Leclerc et al., 1997; Rivlin et al., 2004). This might be one of the reasons for the high abundance of some proteins in vitrified compared to fresh spermatozoa.

Taken together, our laboratory analyses demonstrate that the addition of 0.02 μM MitoQ and 1% glycerol in the basal vitrification medium improves spermatozoa motility. The use of glycerol for spermatozoa vitrification caused a drop in mitochondrial potential and increased oxidative stress, resulting in more DNA fragmentation, less ATP production, and the alteration of more functional proteins. TEM revealed that even a low concentration of a permeable cryoprotectant (1% glycerol) adversely affects the ultrastructure of the spermatozoa head. Still, the addition of 0.02 μM MitoQ protects the ultrastructure of the spermatozoa head from cryo-damage during the vitrification process. Proteomics analysis of the vitrified spermatozoa revealed that the alterations in the proteins responsible for spermatozoa cytoskeleton, phosphorylation, and ubiquitination were low compared to those previously reported for the conventional slow-freezing method. Also, proteomics analysis revealed that MitoQ, but not glycerol, protected from spermatozoa protein modifications responsible for spermatozoa motility, capacitation, acrosome reaction, spermatozoa–egg fusion, spermatozoa penetration, and early embryonic development during the vitrification process. However, we are conscious that our study has some limitations. Our experimental design does not allow distinguishing whether the alterations of the proteins are due to the different vitrification medium compositions or due to vitrification or a combination of both. For this, additional experiments are required to scrutinize our findings as to the action of MitoQ and glycerol on the proteome.

## Conclusion

We demonstrate that MitoQ protects spermatozoa motility, membrane integrity, and ATP production after the vitrification and warming processes. We also demonstrate that MitoQ alleviates oxidative stress induced by the vitrification process. The electron microscopy and proteomics results support our findings that MitoQ supplementation results in minimal ultrastructural and protein alterations during the process.

## Data Availability Statement

The datasets presented in this study can be found in online repositories. The names of the repository/repositories and accession number(s) can be found below: ProteomeXchange with identifier PXD025939.

## Ethics Statement

The studies involving human participants were reviewed and approved by University Review Boards, the University of Cologne (application 01-106). The patients/participants provided their written informed consent to participate in this study.

## Author Contributions

PK, EI, and VI designed the study. PK and MW performed the experiments, wrote the article, and prepared the figures. PK, MW, EI, and VI analyzed and interpreted the data. EI, VI, GR, PM, WW, and MB critically revised the article. PK, WW, and MW contributed statistical expertise. PK, MW, and WW did the literature search. All authors read and approved the final manuscript.

## Conflict of Interest

The authors declare that the research was conducted in the absence of any commercial or financial relationships that could be construed as a potential conflict of interest.
