# Supplementary material for: Unraveling Subcellular and Ultrastructural Changes During Vitrification of Human Spermatozoa: Effect of a Mitochondria-Targeted Antioxidant and a Permeable Cryoprotectant
Source: Front Cell Dev Biol. 2021 Jul 2;9:672862. doi: 10.3389/fcell.2021.672862 (PMC8284099; doi:10.3389/fcell.2021.672862)
Supplement: Supplementary file 2 [file Table_2.DOCX]

**Supplementary Table 2: List of low abundant proteins (n= 118) after sperm vitrification compared to fresh sperm**

| **Protein IDs** | **Gene names** | **Protein names** | **Down-regulated in groups** |
| --- | --- | --- | --- |
| P46783 | RPS10 | 40S ribosomal protein S10 | BM/Fresh  MitoQ/Fresh Glycerol/Fresh  Mito-Gly/Fresh |
| P08708 | RPS17 | 40S ribosomal protein S17 | BM/Fresh  MitoQ/Fresh  Glycerol/Fresh  Mito-Gly/Fresh |
| P39019 | RPS19 | 40S ribosomal protein S19 | BM/Fresh |
| Q8TCD5 | NT5C | 5(3)-deoxyribonucleotidase, cytosolic type | BM/Fresh |
| P62888 | RPL30 | 60S ribosomal protein L30 | BM/Fresh  Glycerol/Fresh |
| P62910 | RPL32 | 60S ribosomal protein L32 | MitoQ/Fresh |
| P83881 | RPL36A | 60S ribosomal protein L36a | MitoQ/Fresh  Mito-Gly/Fresh |
| P62917 | RPL8 | 60S ribosomal protein L8 | BM/Fresh  MitoQ/Fresh  Glycerol/Fresh |
| O95336 | PGLS | 6-phosphogluconolactonase | Glycerol/Fresh |
| Q9Y614 | ACTL7B | Actin-like protein 7B | MitoQ/Fresh, Glycerol/Fresh |
| O14734 | ACOT8 | Acyl-coenzyme A thioesterase 8 | MitoQ/Fresh  Glycerol/Fresh Mito-Gly/Fresh |
| P54819 | AK2 | Adenylate kinase 2, mitochondrial | MitoQ/Fresh |
| P04217 | A1BG | Alpha-1B-glycoprotein | Mito-Gly/Fresh |
| P05067 | APP | Amyloid beta A4 protein | Glycerol/Fresh Mito-Gly/Fresh |
| O43681 | ASNA1 | ATPase ASNA1 | Mito-Gly/Fresh |
| Q92499 | DDX1 | ATP-dependent RNA helicase DDX1 | BM/Fresh  Glycerol/Fresh  Mito-Gly/Fresh |
| O00148 | DDX39A | ATP-dependent RNA helicase DDX39A | BM/Fresh  MitoQ/Fresh  Mito-Gly/Fresh |
| O15523 | DDX3Y | ATP-dependent RNA helicase DDX3Y | Mito-Gly/Fresh |
| P29374 | ARID4A | AT-rich interactive domain-containing protein 4A | BM/Fresh  MitoQ/Fresh  Mito-Gly/Fresh |
| Q9H3K6 | BOLA2 | BolA-like protein 2 | Glycerol/Fresh |
| P11586 | MTHFD1 | C-1-tetrahydrofolate synthase, cytoplasmic; | Glycerol/Fresh Mito-Gly/Fresh |
| Q16566 | CAMK4 | Calcium/calmodulin-dependent protein kinase type IV | Mito-Gly/Fresh |
| Q9Y259 | CHKB | Choline/ethanolamine kinase | MitoQ/Fresh  Glycerol/Fresh |
| Q13185 | CBX3 | Chromobox protein homolog 3 | BM/Fresh  MitoQ/Fresh  Glycerol/Fresh Mito-Gly/Fresh |
| P48444 | ARCN1 | Coatomer subunit delta | BM/Fresh |
| Q13098 | GPS1 | COP9 signalosome complex subunit 1 | MitoQ/Fresh |
| P09669 | COX6C | Cytochrome c oxidase subunit 6C | Glycerol/Fresh Mito-Gly/Fresh |
| Q8IUI8 | CRLF3 | Cytokine receptor-like factor 3 | Glycerol/Fresh |
| Q14204 | DYNC1H1 | Cytoplasmic dynein 1 heavy chain 1 | BM/Fresh  MitoQ/Fresh  Mito-Gly/Fresh |
| Q9Y6G9 | DYNC1LI1 | Cytoplasmic dynein 1 light intermediate chain 1 | Glycerol/Fresh Mito-Gly/Fresh |
| O00273 | DFFA | DNA fragmentation factor subunit alpha | Glycerol/Fresh |
| P31689 | DNAJA1 | DnaJ homolog subfamily A member 1 | BM/Fresh |
| P39656 | DDOST | Dolichyl-diphosphooligosaccharide--protein glycosyltransferase 48 kDa subunit | BM/Fresh |
| Q9P225;Q96DT5 | DNAH2 | Dynein heavy chain 2, axonemal | BM/Fresh  MitoQ/Fresh  Glycerol/Fresh Mito-Gly/Fresh |
| Q9Y4X5 | ARIH1 | E3 ubiquitin-protein ligase ARIH1 | BM/Fresh  MitoQ/Fresh  Glycerol/Fresh |
| Q7Z6Z7 | HUWE1 | E3 ubiquitin-protein ligase HUWE1 | MitoQ/Fresh |
| Q9H4M9 | EHD1 | EH domain-containing protein 1 | BM/Fresh  MitoQ/Fresh  Glycerol/Fresh |
| Q14240 | EIF4A2 | Eukaryotic initiation factor 4A-II | MitoQ/Fresh  Glycerol/Fresh |
| O15371 | EIF3D | Eukaryotic translation initiation factor 3 subunit D | BM/Fresh  MitoQ/Fresh |
| O15372 | EIF3H | Eukaryotic translation initiation factor 3 subunit H | MitoQ/Fresh Glycerol/Fresh |
| Q04637 | EIF4G1 | Eukaryotic translation initiation factor 4 gamma 1 | Glycerol/Fresh |
| P02794 | FTH1 | Ferritin heavy chain | Glycerol/Fresh MitoGlycerol/Fresh |
| P11413 | G6PD | Glucose-6-phosphate 1-dehydrogenase | BM/Fresh |
| Q6IA69 | NADSYN1 | Glutamine-dependent NAD(+) synthetase | BM/Fresh  MitoQ/Fresh  Glycerol/Fresh Mito-Gly/Fresh |
| P46976 | GYG1 | Glycogenin-1 | Mito-Gly/Fresh |
| P49915 | GMPS | GMP synthase [glutamine-hydrolyzing] | BM/Fresh  MitoQ/Fresh  Glycerol/Fresh Mito-Gly/Fresh |
| Q14103 | HNRNPD | Heterogeneous nuclear ribonucleoprotein D0 | Mito-Gly/Fresh |
| Q00839 | HNRNPU | Heterogeneous nuclear ribonucleoprotein U | MitoQ/Fresh  Mito-Gly/Fresh |
| P22626 | HNRNPA2B1 | Heterogeneous nuclear ribonucleoproteins A2/B1 | BM/Fresh Glycerol/Fresh Mito-Gly/Fresh |
| P09429 | HMGB1 | High mobility group protein B1 | Mito-Gly/Fresh |
| P12081 | HARS | Histidine--tRNA ligase, cytoplasmic | Mito-Gly/Fresh |
| Q9UBN7 | HDAC6 | Histone deacetylase 6 | Mito-Gly/Fresh |
| Q71UI9 | H2AFV | Histone H2A.V;Histone H2A.Z | Glycerol/Fresh |
| Q09028 | RBBP4 | Histone-binding protein RBBP4 | BM/Fresh |
| Q9NZL4 | HSPBP1 | Hsp70-binding protein 1 | BM/Fresh  Glycerol/Fresh Mito-Gly/Fresh |
| Q9NPH2 | ISYNA1 | Inositol-3-phosphate synthase 1 | Glycerol/Fresh |
| Q12906 | ILF3 | Interleukin enhancer-binding factor 3 | MitoQ/Fresh  Glycerol/Fresh Mito-Gly/Fresh |
| P09960 | LTA4H | Leukotriene A-4 hydrolase | BM/Fresh  MitoQ/Fresh  Glycerol/Fresh Mito-Gly/Fresh |
| O95573 | ACSL3 | Long-chain-fatty-acid--CoA ligase 3 | Glycerol/Fresh |
| P40121 | CAPG | Macrophage-capping protein | BM/Fresh Mito-Gly/Fresh |
| P50579 | METAP2 | Methionine aminopeptidase 2 | BM/Fresh  Glycerol/Fresh |
| Q9Y6C9 | MTCH2 | Mitochondrial carrier homolog 2 | MitoQ/Fresh  Glycerol/Fresh Mito-Gly/Fresh |
| P35580 | MYH10 | Myosin-10 | Glycerol/Fresh Mito-Gly/Fresh |
| Q9UHY1 | NRBP1 | Nuclear receptor-binding protein | Mito-Gly/Fresh |
| P19338 | NCL | Nucleolin | Mito-Gly/Fresh |
| P22392 | NME2 | Nucleoside diphosphate kinase B | Glycerol/Fresh |
| Q8WXF1 | PSPC1 | Paraspeckle component 1 | BM/Fresh  Mito-Gly/Fresh |
| Q13451 | FKBP5 | Peptidyl-prolyl cis-trans isomerase FKBP5 | Glycerol/Fresh |
| Q13526 | PIN1 | Peptidyl-prolyl cis-trans isomerase NIMA-interacting 1 | Mito-Gly/Fresh |
| P51659 | HSD17B4 | Peroxisomal multifunctional enzyme type 2;(3R)-hydroxyacyl-CoA dehydrogenase;Enoyl-CoA hydratase 2 | Mito-Gly/Fresh |
| Q9Y263 | PLAA | Phospholipase A-2-activating protein | BM/Fresh  Mito-Gly/Fresh |
| O60256 | PRPSAP2 | Phosphoribosyl pyrophosphate synthase-associated protein 2 | BM/Fresh  MitoQ/Fresh  Mito-Gly/Fresh |
| Q9NX46 | ADPRHL2 | Poly(ADP-ribose) glycohydrolase ARH3 | Mito-Gly/Fresh |
| Q15365 | PCBP1 | Poly(rC)-binding protein 1 | BM/Fresh  MitoQ/Fresh  Glycerol/Fresh Mito-Gly/Fresh |
| P11940 | PABPC1 | Polyadenylate-binding protein 1 | BM/Fresh  Mito-Gly/Fresh |
| Q92841 | DDX17 | Probable ATP-dependent RNA helicase DDX17 | Mito/Fresh  Glycerol/Fresh Mito-Gly/Fresh |
| Q9UL46 | PSME2 | Proteasome activator complex subunit 2 | Mito-Gly/Fresh |
| O95456 | PSMG1 | Proteasome assembly chaperone 1 | Glycerol/Fresh |
| Q53FA7 | TP53I3 | Quinone oxidoreductase PIG3 | BM/Fresh  MitoQ/Fresh Glycerol/Fresh |
| O95825 | CRYZL1 | Quinone oxidoreductase-like protein 1 | BM/Fresh  Glycerol/Fresh  Mito-Gly/Fresh |
| Q9NQC3 | RTN4 | Reticulon-4 | Mito-Gly/Fresh |
| Q9Y3A5 | SBDS | Ribosome maturation protein SBDS | MitoQ/Fresh  Glycerol/Fresh |
| P16083 | NQO2 | Ribosyldihydronicotinamide dehydrogenase [quinone] | Mito-Gly/Fresh |
| Q96AT9 | RPE | Ribulose-phosphate 3-epimerase | BM/Fresh |
| P16615 | ATP2A2 | Sarcoplasmic/endoplasmic reticulum calcium ATPase 2 | BM/Fresh  MitoQ/Fresh  Glycerol/Fresh Mito-Gly/Fresh |
| Q9UPN7 | PPP6R1 | Serine/threonine-protein phosphatase 6 regulatory subunit 1 | BM/Fresh  MitoQ/Fresh  Glycerol/Fresh Mito-Gly/Fresh |
| O75170 | PPP6R2 | Serine/threonine-protein phosphatase 6 regulatory subunit 2 | BM/Fresh |
| P49591 | SARS | Serine--tRNA ligase, cytoplasmic | BM/Fresh  MitoQ/Fresh  Glycerol/Fresh Mito-Gly/Fresh |
| O75368 | SH3BGRL | SH3 domain-binding glutamic acid-rich-like protein | Glycerol/Fresh |
| P49458 | SRP9 | Signal recognition particle 9 kDa protein | BM/Fresh  MitoQ/Fresh  Glycerol/Fresh Mito-Gly/Fresh |
| O60749 | SNX2 | Sorting nexin-2 | Mito-Gly/Fresh |
| Q9Y5X1 | SNX9 | Sorting nexin-9 | Glycerol/Fresh |
| Q13813 | SPTAN1 | Spectrin alpha chain, non-erythrocytic 1 | Glycerol/Fresh |
| Q01082 | SPTBN1 | Spectrin beta chain, non-erythrocytic 1 | BM/Fresh  Glycerol/Fresh |
| Q13435 | SF3B2 | Splicing factor 3B subunit 2 | BM/Fresh  Glycerol/Fresh  Mito-Gly/Fresh |
| Q7KZF4 | SND1 | Staphylococcal nuclease domain-containing protein 1 | BM/Fresh  MitoQ/Fresh  Glycerol/Fresh Mito-Gly/Fresh |
| P52888 | THOP1 | Thimet oligopeptidase | Glycerol/Fresh Mito-Gly/Fresh |
| P26639 | TARS | Threonine--tRNA ligase, cytoplasmic | Mito-Gly/Fresh |
| P62328 | TMSB4X | Thymosin beta-4 | Glycerol/Fresh Mito-Gly/Fresh |
| Q00577 | PURA | Transcriptional activator protein Pur-alpha | BM/Fresh |
| P29401 | TKT | Transketolase | Glycerol/Fresh |
| Q9H0I9 | TKTL2 | Transketolase-like protein 2 | BM/Fresh  MitoQ/Fresh  Glycerol/Fresh Mito-Gly/Fresh |
| Q15631 | TSN | Translin | Glycerol/Fresh |
| Q6UW68 | TMEM205 | Transmembrane protein 205 | Glycerol/Fresh |
| Q9NYL9 | TMOD3 | Tropomodulin-3 | MitoQ/Fresh  Glycerol/Fresh Mito-Gly/Fresh |
| P06753 | TPM3 | Tropomyosin alpha-3 chain | Glycerol/Fresh |
| O75347 | TBCA | Tubulin-specific chaperone A | BM/Fresh  MitoQ/Fresh  Glycerol/Fresh Mito-Gly/Fresh |
| O95777 | LSM8 | U6 snRNA-associated Sm-like protein LSm8 | BM/Fresh  MitoQ/Fresh  Mito-Gly/Fresh |
| P49427 | CDC34 | Ubiquitin-conjugating enzyme E2 R1 | Mito-Gly/Fresh |
| O00159 | MYO1C | Unconventional myosin-Ic | MitoQ/Fresh |
| Q9UK41 | VPS28 | Vacuolar protein sorting-associated protein 28 homolog | BM/Fresh  Mito-Gly/Fresh |
| P50552 | VASP | Vasodilator-stimulated phosphoprotein | MitoQ/Fresh Glycerol/Fresh |
| O75396 | SEC22B | Vesicle-trafficking protein SEC22b | BM/Fresh  Glycerol/Fresh Mito-Gly/Fresh |
| Q00341 | HDLBP | Vigilin | Glycerol/Fresh |
| Q9Y277 | VDAC3 | Voltage-dependent anion-selective channel protein 3 | BM/Fresh |
| Q9UI12 | ATP6V1H | V-type proton ATPase subunit H | Glycerol/Fresh |
| P13010 | XRCC5 | X-ray repair cross-complementing protein 5 | BM/Fresh |
| P12956 | XRCC6 | X-ray repair cross-complementing protein 6 | MitoQ/Fresh |
