# Supplementary material for: Unraveling Subcellular and Ultrastructural Changes During Vitrification of Human Spermatozoa: Effect of a Mitochondria-Targeted Antioxidant and a Permeable Cryoprotectant
Source: Front Cell Dev Biol. 2021 Jul 2;9:672862. doi: 10.3389/fcell.2021.672862 (PMC8284099; doi:10.3389/fcell.2021.672862)
Supplement: Supplementary file 3 [file Table_3.DOCX]

**Supplementary Table 3: List of differentially high abundant proteins (n= 21) after sperm vitrification compared to fresh sperm.**

| **Protein IDs** | **Gene names** | **Protein names** | **Up-regulated in groups** |
| --- | --- | --- | --- |
| P08253 | MMP2 | 72 kDa type IV collagenase;PEX | Glycerol/Fresh |
| P04217 | A1BG | Alpha-1B-glycoprotein | Mito-Gly/Fresh |
| P05067 | APP | Amyloid beta A4 protein | Glycerol/Fresh Mito-Gly/Fresh |
| P03950 | ANG | Angiogenin | Mito-Gly/Fresh |
| Q13939 | CCIN | Calicin | Mito-Gly/Fresh |
| Q8WXQ8 | CPA5 | Carboxypeptidase A5 | MitoQ/Fresh  Glycerol/Fresh Mito-Gly/Fresh |
| O14578 | CIT | Citron Rho-interacting kinase | Glycerol/Fresh |
| Q9NZP8 | C1RL | Complement C1r subcomponent-like protein | Glycerol/Fresh |
| P52943 | CRIP2 | Cysteine-rich protein 2 | BM/Fresh Glycerol/Fresh |
| O00115 | DNASE2 | Deoxyribonuclease-2-alpha | BM/Fresh |
| P53634 | CTSC | Dipeptidyl peptidase 1 | BM/Fresh  MitoQ/Fresh  Mito-Gly/Fresh |
| O95967 | EFEMP2 | EGF-containing fibulin-like extracellular matrix protein 2 | Glycerol/Fresh |
| P00738 | HP | Haptoglobin;Haptoglobin alpha chain | Mito-Gly/Fresh |
| P68871 | HBB | Hemoglobin subunit beta, Spinorphin | Mito-Gly/Fresh |
| P24593 | IGFBP5 | Insulin-like growth factor-binding protein 5 | Mito-Gly/Fresh |
| P02753 | RBP4 | Retinol-binding protein 4 | BM/Fresh  MitoQ/Fresh  Mito-Gly/Fresh |
| Q6P4F7 | ARHGAP11A | Rho GTPase-activating protein 11A | BM/Fresh |
| Q9P1V8 | SAMD15 | Sterile alpha motif domain-containing protein 15 | MitoQ/Fresh |
| Q6ZNM6 | TEX43 | Testis-expressed sequence 43 protein | BM/Fresh  MitoQ/Fresh  Glycerol/Fresh Mito-Gly/Fresh |
| O43657 | TSPAN6 | Tetraspanin-6 | Glycerol/Fresh |
| Q9H5F2 | C11orf1 | UPF0686 protein C11orf1 | Glycerol/Fresh |
