# Supplementary material for: Unraveling Subcellular and Ultrastructural Changes During Vitrification of Human Spermatozoa: Effect of a Mitochondria-Targeted Antioxidant and a Permeable Cryoprotectant
Source: Front Cell Dev Biol. 2021 Jul 2;9:672862. doi: 10.3389/fcell.2021.672862 (PMC8284099; doi:10.3389/fcell.2021.672862)
Supplement: Supplementary file 4 [file Table_4.DOCX]

**Supplementary Table 4: List of altered proteins (n= 19) that common to all treatment groups.**

| **Gene Name** | **Protein IDs** | **Protein names** |
| --- | --- | --- |
| DNAH2 | Q9P225 | Dynein heavy chain 2 |
| CTSH | P09668 | Pro-cathepsin H |
| HSPG2 | P98160 | Basement membrane-specific heparan sulfate proteoglycan core protein |
| RPS17 | P08708 | 40S ribosomal protein S17 |
| PCBP1 | Q15365 | Poly(rC) binding protein 1 |
| SND1 | Q7KZF4 | Staphylococcal nuclease domain-containing protein 1 |
| TLN1 | Q9Y490 | Talin-1 |
| CBX3 | Q13185 | Chromobox protein homolog 3 |
| SARS | P49591 | Serine-tRNA ligase |
| SRP9 | P49458 | Signal recognition particle 9 kDa protein |
| GMPS | P49915 | GMP synthase [glutamine-hydrolyzing] |
| RPS10 | P46783 | 40S ribosomal protein S10 |
| ATP2A2 | P16615 | Sarcoplasmic/endoplasmic reticulum calcium ATPase 2 |
| PPP6R1 | Q9UPN7 | Serine/threonine-protein phosphatase 6 regulatory subunit 1 |
| TKTL2 | Q9H0I9 | Transketolase-like protein 2 |
| NADSYN1 | Q6IA69 | Glutamine-dependent NAD(+) synthetase |
| LTA4H | P09960 | Leukotriene A-4 hydrolase |
| TEX43 | Q6ZNM6 | Testis-expressed sequence 43 protein |
| TBCA | O75347 | Tubulin-specific chaperone A |

**Supplementary Table 5: List of altered proteins (n= 17) identified exclusively in Basal medium group.**

| **Gene Name** | **Protein IDs** | **Protein names** |
| --- | --- | --- |
| DNAJA1 | P31689 | DnaJ homolog subfamily A member 1 |
| PURA | Q00577 | Transcriptional activator protein Pur-alpha |
| NT5C | Q8TCD5 | 5(3)-deoxyribonucleotidase |
| DNASE2 | O00115 | Deoxyribonuclease-2-alpha |
| RBBP4 | Q09028 | Histone-binding protein RBBP4 |
| ARHGAP11A | Q6P4F7 | Rho GTPase-activating protein 11A |
| RPE | Q96AT9 | Ribulose-phosphate 3-epimerase |
| SYAP1 | Q96A49 | Synapse-associated protein 1 |
| ARCN1 | P48444 | Coatomer subunit delta |
| PPP6R2 | O75170 | Serine/threonine-protein phosphatase 6 regulatory subunit 2 |
| DDOST | P39656 | Dolichyl-diphosphooligosaccharide-protein glycosyltransferase 48 kDa subunit |
| SETSIP | Q01105 | Protein SET;Protein SETSIP |
| XRCC5 | P13010 | X-ray repair cross-complementing protein 5 |
| RPS19 | P39019 | 40S ribosomal protein S19 |
| G6PD | P11413 | Glucose-6-phosphate 1-dehydrogenase |
| VDAC3 | Q9Y277 | Voltage-dependent anion-selective channel protein 3 |
| SMOC2 | Q9H3U7 | SPARC-related modular calcium-binding protein 2 |

**Supplementary Table 6: List of altered proteins (n= 8) identified exclusively in Mito Q group.**

| Gene Name | Protein IDs | Protein names |
| --- | --- | --- |
| XRCC6 | P12956 | X-ray repair cross-complementing protein 6 |
| RPS27 | P42677 | 40S ribosomal protein S27 |
| AK2 | P54819 | Adenylate kinase 2; mitochondrial |
| RPL32 | P62910 | 60S ribosomal protein L32 |
| GPS1 | Q13098 | COP9 signalosome complex subunit 1 |
| HUWE1 | Q7Z6Z7 | E3 ubiquitin-protein ligase HUWE1 |
| SAMD15 | Q9P1V8 | Sterile alpha motif domain-containing protein 15 |
| MYO1C | O00159 | Unconventional myosin-Ic |

**Supplementary Table 7: List of altered proteins (n= 32) identified exclusively in glycerol group.**

| **Gene Name** | **Protein IDs** | **Protein names** |
| --- | --- | --- |
| DFFA | O00273 | DNA fragmentation factor subunit alpha |
| CIT | O14578 | Citron Rho-interacting kinase |
| SH3BGRL | O75368 | SH3 domain-binding glutamic acid-rich-like protein |
| PGLS | O95336 | 6-phosphogluconolactonase |
| PSMG1 | O95456 | Proteasome assembly chaperone 1 |
| ACSL3 | O95573 | Long-chain-fatty-acid--CoA ligase 3 |
| FN1 | P02751 | Fibronectin;Anastellin;Ugl-Y1;Ugl-Y2;Ugl-Y3 |
| TPM3 | P06753 | Tropomyosin alpha-3 chain |
| H2AFV | Q71UI9 | Histone H2A.V;Histone H2A.Z |
| NME2 | P22392 | Nucleoside diphosphate kinase B;Putative nucleoside diphosphate kinase |
| TKT | P29401 | Transketolase |
| FNTA | P49354 | Protein farnesyltransferase/ geranylgeranyltransferase type-1 subunit alpha |
| HDLBP | Q00341 | Vigilin |
| EIF4G1 | Q04637 | Eukaryotic translation initiation factor 4 gamma 1 |
| FKBP5 | Q13451 | Peptidyl-prolyl cis-trans isomerase FKBP5;Peptidyl-prolyl cis-trans isomerase FKBP5 |
| SPTAN1 | Q13813 | Spectrin alpha chain |
| TSN | Q15631 | Translin |
| TMEM205 | Q6UW68 | Transmembrane protein 205 |
| BOLA2 | Q9H3K6 | BolA-like protein 2 |
| C11orf1 | Q9H5F2 | UPF0686 protein C11orf1 |
| ISYNA1 | Q9NPH2 | Inositol-3-phosphate synthase 1 |
| LANCL2 | Q9NS86 | LanC-like protein 2 |
| ATP6V1H | Q9UI12 | V-type proton ATPase subunit H |
| SNX9 | Q9Y5X1 | Sorting nexin-9 |
| TSPAN6 | O43657 | Tetraspanin-6 |
| EFEMP2 | O95967 | EGF-containing fibulin-like extracellular matrix protein 2 |
| MMP2 | P08253 | 72 kDa type IV collagenase;PEX |
| KHDRBS1 | Q07666 | KH domain-containing RNA-binding signal transduction-associated protein 1 |
| FAM209A | Q5JX71 | Protein FAM209A |
| C1RL | Q9NZP8 | Complement C1r subcomponent-like protein |
| CFAP70 | Q5T0N1 | Cilia- and flagella-associated protein 70 |
| APP | P05067 | Amyloid beta A4 protein |

**Supplementary Table 8: List of altered proteins (n= 24) identified only in Mito-Gly group.**

| Gene Name | Protein IDs | Protein names |
| --- | --- | --- |
| ASNA1 | O43681 | ATPase ASNA1 |
| HMGB1 | P09429 | High mobility group protein B1 |
| NQO2 | P16083 | Ribosyldihydronicotinamide dehydrogenase [quinone] |
| TARS | P26639 | Threonine--tRNA ligase |
| GYG1 | P46976 | Glycogenin-1 |
| CDC34 | P49427;Q712K3 | Ubiquitin-conjugating enzyme E2 R1 |
| RPL14 | P50914 | 60S ribosomal protein L14 |
| HSD17B4 | P51659 | Peroxisomal multifunctional enzyme type 2;(3R)-hydroxyacyl-CoA dehydrogenase;Enoyl-CoA hydratase 2 |
| RPL18A | Q02543 | 60S ribosomal protein L18a |
| PIN1 | Q13526 | Peptidyl-prolyl cis-trans isomerase NIMA-interacting 1 |
| HNRNPD | Q14103 | Heterogeneous nuclear ribonucleoprotein D0 |
| CAMK4 | Q16566 | Calcium/calmodulin-dependent protein kinase type IV |
| STX12 | Q86Y82 | Syntaxin-12 |
| RTN4 | Q9NQC3 | Reticulon-4 |
| ADPRHL2 | Q9NX46 | Poly(ADP-ribose) glycohydrolase ARH3 |
| HDAC6 | Q9UBN7 | Histone deacetylase 6 |
| NRBP1 | Q9UHY1 | Nuclear receptor-binding protein |
| PSME2 | Q9UL46 | Proteasome activator complex subunit 2 |
| HP | P00738 | Haptoglobin;Haptoglobin alpha chain;Haptoglobin beta chain |
| ANG | P03950 | Angiogenin |
| A1BG | P04217 | Alpha-1B-glycoprotein |
| IGFBP5 | P24593 | Insulin-like growth factor-binding protein 5 |
| HBB | P68871;P02042 | Hemoglobin subunit beta;LVV-hemorphin-7;Spinorphin |
| CCIN | Q13939 | Calicin |
