# Supplementary material for: Unraveling Subcellular and Ultrastructural Changes During Vitrification of Human Spermatozoa: Effect of a Mitochondria-Targeted Antioxidant and a Permeable Cryoprotectant
Source: Front Cell Dev Biol. 2021 Jul 2;9:672862. doi: 10.3389/fcell.2021.672862 (PMC8284099; doi:10.3389/fcell.2021.672862)
Supplement: Supplementary file 5 [file Table_5.DOCX]

**Supplementary Table 9: Enrichment Analysis: Differentially altered proteins after vitrification with respect to cellular components of sperm.**

| **Cellular Component** | **Total annotated genes in this class** | **No of proteins identified in sperm** | **No of proteins differentially expressed after vitrification** | | | |
| --- | --- | --- | --- | --- | --- | --- |
|  |  |  | **Basal medium** | **Mito Q** | **T3 Glycerol** | **Mito-Gly** |
| Cytoplasm | 5684 | 994 | 40 | 36 | 52 | 55 |
| Exosomes | 2043 | 762 | 24 | 19 | 34 | 26 |
| Nucleus | 5847 | 636 | 31 | 22 | 35 | 38 |
| Lysosome | 1620 | 586 | 17 | 8 | 18 | 14 |
| Cytosol | 1178 | 353 | 16 | 17 | 23 | 20 |
| Mitochondrion | 1259 | 344 | 13 | 10 | 12 | 18 |
| Plasma membrane | 3479 | 300 | 6 | 7 | 15 | 12 |
| Centrosome | 656 | 267 | 17 | 12 | 16 | 18 |
| Nucleolus | 1257 | 236 | 20 | 17 | 18 | 26 |
| Cytoskeleton | 427 | 116 | 7 | 7 | 10 | 7 |
| Membrane | 350 | 65 | 2 | 1 | 3 | 3 |
| Microtubule | 129 | 36 | 0 | 0 | 1 | 1 |
| Ribonucleoprotein complex | 65 | 36 | 4 | 3 | 3 | 7 |
| Actin cytoskeleton | 132 | 34 | 0 | 3 | 4 | 1 |
| Microsome | 156 | 21 | 2 | 1 | 1 | 1 |
| Intracellular membrane-bounded organelle | 127 | 21 | 3 | 1 | 2 | 2 |
| Perinuclear region | 131 | 20 | 0 | 0 | 1 | 0 |
| Nuclear membrane | 78 | 19 | 1 | 0 | 0 | 1 |
| Protein complex | 65 | 13 | 1 | 0 | 0 | 0 |
| Microtubule cytoskeleton | 47 | 12 | 0 | 0 | 1 | 0 |
| Mitochondrial outer membrane | 29 | 9 | 1 | 0 | 0 | 0 |
| Clathrin-coated vesicle | 19 | 6 | 0 | 0 | 1 | 0 |
| Stress fiber | 16 | 6 | 0 | 0 | 2 | 1 |
| Microtubule associated complex | 24 | 6 | 0 | 0 | 1 | 1 |
| Eukaryotic translation initiation factor 4F complex | 9 | 5 | 0 | 1 | 2 | 0 |
| Spliceosomal complex | 46 | 5 | 2 | 0 | 2 | 2 |
| Ubiquitin ligase complex | 43 | 4 | 1 | 1 | 1 | 0 |
| Acrosome | 11 | 4 | 1 | 0 | 0 | 0 |
| F-actin capping protein complex | 6 | 4 | 1 | 0 | 0 | 1 |
| NuRD complex | 12 | 3 | 1 | 0 | 0 | 0 |
| Myosin complex | 10 | 3 | 0 | 0 | 1 | 1 |
| Cytoplasmic dynein complex | 6 | 3 | 1 | 1 | 1 | 2 |
| Spectrin | 9 | 2 | 1 | 0 | 2 | 0 |
| Fibrinogen complex | 7 | 2 | 0 | 0 | 1 | 0 |
| Muscle thin filament tropomyosin | 4 | 2 | 0 | 0 | 1 | 0 |
| Nuclear chromosome, telomeric region | 12 | 1 | 1 | 0 | 0 | 0 |
| COPII vesicle coat | 5 | 1 | 1 | 0 | 1 | 1 |
| Intracellular ferritin complex | 2 | 1 | 0 | 0 | 1 | 1 |
| Stress granule | 8 | 1 | 1 | 0 | 1 | 1 |
| Histone deacetylase complex | 21 | 1 | 0 | 0 | 0 | 1 |
| Dynein complex | 2 | 1 | 0 | 0 | 0 | 1 |
