# Supplementary material for: Unraveling Subcellular and Ultrastructural Changes During Vitrification of Human Spermatozoa: Effect of a Mitochondria-Targeted Antioxidant and a Permeable Cryoprotectant
Source: Front Cell Dev Biol. 2021 Jul 2;9:672862. doi: 10.3389/fcell.2021.672862 (PMC8284099; doi:10.3389/fcell.2021.672862)
Supplement: Supplementary file 6 [file Table_6.DOCX]

**Supplementary Table 10. Enrichment Analysis: Number of differentially altered proteins after vitrification with respect to molecular functions of sperm.**

| **Molecular Function** | **Total annotated proteins in this class** | **No of proteins identified in sperm** | **No of proteins differentially down regulated after vitrification** | | | |
| --- | --- | --- | --- | --- | --- | --- |
|  |  |  | **Basal medium** | **Mito Q** | **T3 Glycerol** | **Mito-Gly** |
| Transporter activity | 576 | 103 | 4 | 2 | 3 | 5 |
| Hydrolase activity | 203 | 55 | 2 | 2 | 3 | 3 |
| Chaperone activity | 126 | 48 | 1 | 1 | 2 | 1 |
| Oxidoreductase activity | 161 | 46 | 2 | 1 | 3 | 3 |
| RNA binding | 366 | 40 | 8 | 4 | 6 | 13 |
| Ligase activity | 112 | 38 | 2 | 2 | 2 | 5 |
| Calcium ion binding | 185 | 37 | 2 | 2 | 2 | 0 |
| Structural constituent of cytoskeleton | 137 | 33 | 1 | 1 | 3 | 0 |
| Structural molecule activity | 269 | 31 | 0 | 0 | 1 | 1 |
| Receptor signaling complex scaffold activity | 322 | 29 | 2 | 0 | 1 | 2 |
| Cytoskeletal protein binding | 218 | 27 | 2 | 3 | 5 | 4 |
| Isomerase activity | 45 | 21 | 0 | 0 | 2 | 1 |
| ATPase activity | 111 | 21 | 2 | 3 | 3 | 4 |
| Protein serine/threonine kinase activity | 301 | 21 | 0 | 0 | 1 | 1 |
| Motor activity | 79 | 20 | 0 | 0 | 1 | 1 |
| Metallopeptidase activity | 101 | 13 | 0 | 0 | 1 | 1 |
| Heat shock protein activity | 27 | 12 | 1 | 0 | 0 | 0 |
| Transcription factor activity | 842 | 11 | 1 | 1 | 2 | 1 |
| Cysteine-type peptidase activity | 46 | 9 | 1 | 0 | 0 | 0 |
| Galactosyltransferase activity | 38 | 4 | 1 | 0 | 0 | 0 |
| Voltage-gated ion channel activity | 130 | 3 | 1 | 0 | 0 | 0 |
| DNA repair protein | 57 | 2 | 1 | 0 | 0 | 0 |
| Racemase and epimerase activity | 11 | 2 | 1 | 0 | 0 | 0 |
| Helicase activity | 25 | 2 | 1 | 0 | 0 | 1 |
| Transcription factor binding | 10 | 1 | 1 | 0 | 1 | 1 |
