# Supplementary material for: Unraveling Subcellular and Ultrastructural Changes During Vitrification of Human Spermatozoa: Effect of a Mitochondria-Targeted Antioxidant and a Permeable Cryoprotectant
Source: Front Cell Dev Biol. 2021 Jul 2;9:672862. doi: 10.3389/fcell.2021.672862 (PMC8284099; doi:10.3389/fcell.2021.672862)
Supplement: Supplementary file 7 [file Table_7.DOCX]

**Supplementary Table 11: Enrichment Analysis: Number of differentially altered proteins after vitrification with respect to biological process of sperm.**

| **Biological Process** | **Total annotated proteins in this class** | **No of proteins identified in sperm** | **No of proteins differentially down regulated after vitrification** | | | |
| --- | --- | --- | --- | --- | --- | --- |
|  |  |  | **Basal medium** | **Mito Q** | **T3 Glycerol** | **Mito-Gly** |
| Metabolism | 1683 | 388 | 9 | 6 | 12 | 15 |
| Energy pathways | 1633 | 382 | 9 | 6 | 12 | 15 |
| Protein metabolism | 1323 | 349 | 15 | 13 | 15 | 13 |
| Signal transduction | 3934 | 266 | 7 | 6 | 10 | 6 |
| Cell communication | 3713 | 248 | 6 | 5 | 9 | 5 |
| Cell growth and/or maintenance | 1125 | 154 | 3 | 3 | 10 | 6 |
| Regulation of nucleobase, nucleoside, nucleotide and nucleic acid metabolism | 2828 | 119 | 16 | 11 | 10 | 21 |
| Transport | 1215 | 114 | 4 | 3 | 5 | 7 |
| Cytoskeleton organization and biogenesis | 21 | 5 | 0 | 1 | 1 | 0 |
| Lipid metabolism | 33 | 4 | 0 | 1 | 1 | 1 |
