# Supplementary material for: Unraveling Subcellular and Ultrastructural Changes During Vitrification of Human Spermatozoa: Effect of a Mitochondria-Targeted Antioxidant and a Permeable Cryoprotectant
Source: Front Cell Dev Biol. 2021 Jul 2;9:672862. doi: 10.3389/fcell.2021.672862 (PMC8284099; doi:10.3389/fcell.2021.672862)
Supplement: Supplementary file 8 [file Table_8.DOCX]

**Supplementary Table 12: List of actin and actin-associated proteins identified in sperm and showing differentially altered proteins after vitrification**

| **Protein IDs** | **Gene names** | **Protein names** | **DAPs** |
| --- | --- | --- | --- |
| P68133 | ACTA1 | Actin | NS |
| P63261 | ACTG1 | Actin, cytoplasmic 2 | NS |
| Q9Y615 | ACTL7A | Actin-like protein 7A | NS |
| Q9Y614 | ACTL7B | Actin-like protein 7B | Mito Q/ Fresh  Glycerol/Fresh |
| Q8TC94 | ACTL9 | Actin-like protein 9 | NS |
| Q9NZ32 | ACTR10 | Actin-related protein 10 | NS |
| P61160 | ACTR2 | Actin-related protein 2 | NS |
| Q92747 | ARPC1A | Actin-related protein 2/3 complex subunit 1A | NS |
| O15143 | ARPC1B | Actin-related protein 2/3 complex subunit 1B | NS |
| O15144 | ARPC2 | Actin-related protein 2/3 complex subunit 2 | NS |
| O15145 | ARPC3 | Actin-related protein 2/3 complex subunit 3 | NS |
| P59998 | ARPC4 | Actin-related protein 2/3 complex subunit 4 | NS |
| P61158 | ACTR3 | Actin-related protein 3 | NS |
| Q8TDY3 | ACTRT2 | Actin-related protein T2 | NS |
| Q9BYD9 | ACTRT3 | Actin-related protein T3 | NS |
| P12814 | ACTN1 | Alpha-actinin-1 | NS |
| O43707 | ACTN4 | Alpha-actinin-4 | NS |
| O75882 | ATRN | Attractin | NS |
| P42025 | ACTR1B | Beta-centractin | NS |
| Q14203 | DCTN1 | Dynactin subunit 1 | NS |
| Q13561 | DCTN2 | Dynactin subunit 2 | NS |
| O75935 | DCTN3 | Dynactin subunit 3 | NS |
| Q9UJW0 | DCTN4 | Dynactin subunit 4 | NS |
| Q6ZR08 | DNAH12 | Dynein heavy chain 12, axonemal | NS |
| Q9UFH2 | DNAH17 | Dynein heavy chain 17, axonemal | NS |
| Q9P225 | DNAH2 | Dynein heavy chain 2, axonemal | BM/Fresh  Mito Q/Fresh  Glycerol/Fresh  Mito-Gly/Fresh |
| Q8WXX0 | DNAH7 | Dynein heavy chain 7, axonemal | NS |
| Q96JB1 | DNAH8 | Dynein heavy chain 8, axonemal | NS |
| Q9UI46 | DNAI1 | Dynein intermediate chain 1, axonemal | NS |
| Q9GZS0 | DNAI2 | Dynein intermediate chain 2, axonemal | NS |
| Q4LDG9 | DNAL1 | Dynein light chain 1, axonemal | NS |
| P63167 | DYNLL1 | Dynein light chain 1, cytoplasmic | NS |
| Q96FJ2 | DYNLL2 | Dynein light chain 2, cytoplasmic | NS |
| Q8TF09 | DYNLRB2 | Dynein light chain roadblock-type 2 | NS |
| P63172 | DYNLT1 | Dynein light chain Tctex-type 1 | NS |
| P52907 | CAPZA1 | F-actin-capping protein subunit alpha-1 | NS |
| P47755 | CAPZA2 | F-actin-capping protein subunit alpha-2 | NS |
| Q96KX2 | CAPZA3 | F-actin-capping protein subunit alpha-3 | NS |
| P47756 | CAPZB | F-actin-capping protein subunit beta | NS |
| O14645 | DNALI1 | Axonemal dynein light intermediate polypeptide 1 | NS |
| Q14204 | DYNC1H1 | Cytoplasmic dynein 1 heavy chain 1 | BM/Fresh  Mito Q/Fresh  Mito-Gly/Fresh |
| Q13409 | DYNC1I2 | Cytoplasmic dynein 1 intermediate chain 2 |  |
| Q9Y6G9 | DYNC1LI1 | Cytoplasmic dynein 1 light intermediate chain 1 | Glycerol/Fresh  Mito-Gly/Fresh |
| Q8NCM8 | DYNC2H1 | Cytoplasmic dynein 2 heavy chain 1 | NS |
| Q14203 | DCTN1 | Dynactin subunit 1 | NS |
| Q13561 | DCTN2 | Dynactin subunit 2 | NS |
| O75935 | DCTN3 | Dynactin subunit 3 | NS |
| Q9UJW0 | DCTN4 | Dynactin subunit 4 | NS |
| O00429 | DNM1L | Dynamin-1-like protein | NS |
| P50570 | DNM2 | Dynamin-2 | NS |
| Q6ZR08 | DNAH12 | Dynein heavy chain 12, axonemal | NS |
| Q9UFH2 | DNAH17 | Dynein heavy chain 17, axonemal | NS |
| Q9P225 | DNAH2 | Dynein heavy chain 2, axonemal | BM/Fresh  Mito Q/Fresh  Glycerol/Fresh  Mito-Gly/Fresh |
| Q8WXX0 | DNAH7 | Dynein heavy chain 7, axonemal | NS |
| Q96JB1 | DNAH8 | Dynein heavy chain 8, axonemal | NS |
| Q9UI46 | DNAI1 | Dynein intermediate chain 1, axonemal | NS |
| Q9GZS0 | DNAI2 | Dynein intermediate chain 2, axonemal | NS |
| Q4LDG9 | DNAL1 | Dynein light chain 1, axonemal | NS |
| P63167 | DYNLL1 | Dynein light chain 1, cytoplasmic | NS |
| Q96FJ2 | DYNLL2 | Dynein light chain 2, cytoplasmic | NS |
| Q8TF09 | DYNLRB2 | Dynein light chain roadblock-type 2 | NS |
| P63172 | DYNLT1 | Dynein light chain Tctex-type 1 | NS |
| Q13813 | SPTAN1 | Spectrin alpha chain, non-erythrocytic 1 | Glycerol/Fresh |
| Q01082 | SPTBN1 | Spectrin beta chain, non-erythrocytic 1 | BM/Fresh  Glycerol/Fresh |
| P04632 | CAPNS1 | Calpain small subunit 1 | NS |
| P07384 | CAPN1 | Calpain-1 catalytic subunit | NS |
| P17655 | CAPN2 | Calpain-2 catalytic subunit | NS |
| P18206 | VCL | Vinculin | NS |
| Q9Y490 | TLN1 | Talin-1 | BM/Fresh  Mito Q/Fresh  Glycerol/Fresh  Mito-Gly/Fresh |
| Q9NYL9 | TMOD3 | Tropomodulin-3 | Mito Q/Fresh  Glycerol/Fresh  Mito-Gly/Fresh |
| P06753 | TPM3 | Tropomyosin alpha-3 chain | Glycerol/Fresh |
| P67936 | TPM4 | Tropomyosin alpha-4 chain | NS |
| P50552 | VASP | Vasodilator-stimulated phosphoprotein | Mito Q/Fresh  Glycerol/Fresh |
| P50552 | VASP | Vasodilator-stimulated phosphoprotein | Mito Q/Fresh  Glycerol/Fresh |
| P07737 | PFN1 | Profilin-1 | NS |
| P35080 | PFN2 | Profilin-2 | NS |
