# Supplementary material for: Unraveling Subcellular and Ultrastructural Changes During Vitrification of Human Spermatozoa: Effect of a Mitochondria-Targeted Antioxidant and a Permeable Cryoprotectant
Source: Front Cell Dev Biol. 2021 Jul 2;9:672862. doi: 10.3389/fcell.2021.672862 (PMC8284099; doi:10.3389/fcell.2021.672862)
Supplement: Supplementary file 9 [file Table_9.DOCX]

**Supplementary Table 13: List of tubulins identified in sperm and showing differentially altered proteins (DAPs) after vitrification**

| **Protein IDs** | **Gene names** | **Protein names** | **DAPs** |
| --- | --- | --- | --- |
| Q71U36 | TUBA1A | Tubulin alpha-1A chain | NS |
| P68363 | TUBA1B | Tubulin alpha-1B chain | NS |
| Q9BQE3 | TUBA1C | Tubulin alpha-1C chain | NS |
| P0DPH8 | TUBA3E | Tubulin alpha-3E chain | NS |
| P68366 | TUBA4A | Tubulin alpha-4A chain | NS |
| P07437 | TUBB | Tubulin beta chain | NS |
| Q13885 | TUBB2A | Tubulin beta-2A chain | NS |
| P68371 | TUBB4B | Tubulin beta-4B chain;Tubulin beta-4A chain | NS |
| Q3ZCM7 | TUBB8 | Tubulin beta-8 chain | NS |
| P59282 | TPPP2 | Tubulin polymerization-promoting protein family member 2 | NS |
| Q99426 | TBCB | Tubulin-folding cofactor B | NS |
| O75347 | TBCA | Tubulin-specific chaperone A | BM/Fresh  Mito Q/Fresh  Glycerol/Fresh  Mito-Gly/Fresh |
| Q9BTW9 | TBCD | Tubulin-specific chaperone D | NS |
| Q14166 | TTLL12 | Tubulin--tyrosine ligase-like protein 12 | NS |
| P59282 | TPPP2 | Tubulin polymerization-promoting protein family member 2 | NS |
