# Supplementary material for: Unraveling Subcellular and Ultrastructural Changes During Vitrification of Human Spermatozoa: Effect of a Mitochondria-Targeted Antioxidant and a Permeable Cryoprotectant
Source: Front Cell Dev Biol. 2021 Jul 2;9:672862. doi: 10.3389/fcell.2021.672862 (PMC8284099; doi:10.3389/fcell.2021.672862)
Supplement: Supplementary file 10 [file Table_10.DOCX]

**Supplementary Table 14: List of ubiquitins, ubiquitinating- and deubiquitinating enzymes, and showing differentially altered proteins (DAPs) after vitrification**

| **Protein IDs** | **Gene names** | **Protein names** | **DAPs** |
| --- | --- | --- | --- |
| Q9Y4X5 | ARIH1 | E3 ubiquitin-protein ligase ARIH1 | BM/Fresh  Mito Q/Fresh  Glycerol/Fresh |
| Q7Z6Z7 | HUWE1 | E3 ubiquitin-protein ligase HUWE1 | Mito Q/Fresh |
| P62877 | RBX1 | E3 ubiquitin-protein ligase RBX1;E3 ubiquitin-protein ligase RBX1, N-terminally processed | NS |
| Q9NQ86 | TRIM36 | E3 ubiquitin-protein ligase TRIM36 | NS |
| Q5T4S7 | UBR4 | E3 ubiquitin-protein ligase UBR4 | NS |
| Q8N9V2 | TRIML1 | Probable E3 ubiquitin-protein ligase TRIML1 | NS |
| Q16186 | ADRM1 | Proteasomal ubiquitin receptor ADRM1 | NS |
| Q8WUN7 | UBTD2 | Ubiquitin domain-containing protein 2 | NS |
| Q92890 | UFD1L | Ubiquitin fusion degradation protein 1 homolog | NS |
| Q96FW1 | OTUB1 | Ubiquitin thioesterase OTUB1 | NS |
| Q96DC9 | OTUB2 | Ubiquitin thioesterase OTUB2 | NS |
| P62979 | RPS27A | Ubiquitin-40S ribosomal protein S27a | NS |
| P62837 | UBE2D2 | Ubiquitin-conjugating enzyme E2 D2;Ubiquitin-conjugating enzyme E2 D3 | NS |
| P61088 | UBE2N | Ubiquitin-conjugating enzyme E2 N;Putative ubiquitin-conjugating enzyme E2 N-like | NS |
| P49427 | CDC34 | Ubiquitin-conjugating enzyme E2 R1 | Mito-Gly/Fresh |
| Q13404 | UBE2V1 | Ubiquitin-conjugating enzyme E2 variant 1 | NS |
| P22314 | UBA1 | Ubiquitin-like modifier-activating enzyme 1 | NS |
| Q9GZZ9 | UBA5 | Ubiquitin-like modifier-activating enzyme 5 | NS |
| A0AVT1 | UBA6 | Ubiquitin-like modifier-activating enzyme 6 | NS |
| Q8N7F7 | UBL4B | Ubiquitin-like protein 4B | NS |
| **Deubiquitinating enzymes** | | |  |
| P54578 | USP14 | Ubiquitin carboxyl-terminal hydrolase 14 | NS |
| Q13107 | USP4 | Ubiquitin carboxyl-terminal hydrolase 4 | NS |
| P45974 | USP5 | Ubiquitin carboxyl-terminal hydrolase 5 | NS |
| Q93009 | USP7 | Ubiquitin carboxyl-terminal hydrolase 7 | NS |
| P09936 | UCHL1 | Ubiquitin carboxyl-terminal hydrolase isozyme L1 | NS |
| P15374 | UCHL3 | Ubiquitin carboxyl-terminal hydrolase isozyme L3 | NS |
| Q9Y5K5 | UCHL5 | Ubiquitin carboxyl-terminal hydrolase isozyme L5 | NS |
| **Subunit composition of the canonical 26S proteasome** | | |  |
| **19S complex non-ATPase subunits** | | |  |
| Q99460 | PSMD1 | 26S proteasome non-ATPase regulatory subunit 1 | NS |
| Q13200 | PSMD2 | 26S proteasome non-ATPase regulatory subunit 2 | NS |
| O43242 | PSMD3 | 26S proteasome non-ATPase regulatory subunit 3 | NS |
| P55036 | PSMD4 | 26S proteasome non-ATPase regulatory subunit 4 | NS |
| Q16401 | PSMD5 | 26S proteasome non-ATPase regulatory subunit 5 | NS |
| Q15008 | PSMD6 | 26S proteasome non-ATPase regulatory subunit 6 | NS |
| P51665 | PSMD7 | 26S proteasome non-ATPase regulatory subunit 7 | NS |
| P48556 | PSMD8 | 26S proteasome non-ATPase regulatory subunit 8 | NS |
| O00233 | PSMD9 | 26S proteasome non-ATPase regulatory subunit 9 | NS |
| O00231 | PSMD11 | 26S proteasome non-ATPase regulatory subunit 11 | NS |
| O00232 | PSMD12 | 26S proteasome non-ATPase regulatory subunit 12 | NS |
| Q9UNM6 | PSMD13 | 26S proteasome non-ATPase regulatory subunit 13 | NS |
| O00487 | PSMD14 | 26S proteasome non-ATPase regulatory subunit 14 | NS |
| **19S complex ATPase subunits** | | |  |
| P62191 | PSMC1 | 26S protease regulatory subunit 4 | NS |
| P35998 | PSMC2 | 26S protease regulatory subunit 7 | NS |
| P17980 | PSMC3 | 26S protease regulatory subunit 6A | NS |
| P43686 | PSMC4 | 26S protease regulatory subunit 6B | NS |
| P62195 | PSMC5 | 26S protease regulatory subunit 8 | NS |
| P62333 | PSMC6 | 26S protease regulatory subunit 10B | NS |
| **20S core α-subunits** | | |  |
| P25786 | PSMA1 | Proteasome subunit alpha type-1 | NS |
| P25787 | PSMA2 | Proteasome subunit alpha type-2 | NS |
| P25788 | PSMA3 | Proteasome subunit alpha type-3 | NS |
| P25789 | PSMA4 | Proteasome subunit alpha type-4 | NS |
| P28066 | PSMA5 | Proteasome subunit alpha type-5 | NS |
| P60900 | PSMA6 | Proteasome subunit alpha type-6 | NS |
| O14818 | PSMA7 | Proteasome subunit alpha type-7 | NS |
| Q8TAA3 | PSMA8 | Proteasome subunit alpha type-7-like | NS |
| **Constitutive 20S core β-subunits** | | |  |
| P20618 | PSMB1 | Proteasome subunit beta type-1 | NS |
| P49721 | PSMB2 | Proteasome subunit beta type-2 | NS |
| P49720 | PSMB3 | Proteasome subunit beta type-3 | NS |
| P28070 | PSMB4 | Proteasome subunit beta type-4 | NS |
| P28074 | PSMB5 | Proteasome subunit beta type-5 | NS |
| P28072 | PSMB6 | Proteasome subunit beta type-6 | NS |
| Q99436 | PSMB7 | Proteasome subunit beta type-7 | NS |
| **Proteasome activator PA200** | | |  |
| Q14997 | PSME4 | Proteasome activator complex subunit 4 | NS |
| **11S complex (PA28)** | | |  |
| Q06323 | PSME1 | Proteasome activator complex subunit 1 |  |
| Q9UL46 | PSME2 | Proteasome activator complex subunit 2 | Mito-Gly/Fresh |
|  |  |  |  |
| O95456 | PSMG1 | Proteasome assembly chaperone 1 | Glycerol/Fresh |
| Q92530 | PSMF1 | Proteasome inhibitor PI31 subunit | NS |
