# Supplementary material for: Unraveling Subcellular and Ultrastructural Changes During Vitrification of Human Spermatozoa: Effect of a Mitochondria-Targeted Antioxidant and a Permeable Cryoprotectant
Source: Front Cell Dev Biol. 2021 Jul 2;9:672862. doi: 10.3389/fcell.2021.672862 (PMC8284099; doi:10.3389/fcell.2021.672862)
Supplement: Supplementary file 11 [file Table_11.DOCX]

**Supplementary Table 15: List of identified proteins in sperm responsible for phosphorylation (kinases) and dephosphorylation (phosphatases) and showing differentially altered proteins (DAPs) after vitrification.**

| **Protein IDs** | **Gene names** | **Protein names** | **DAPs** |
| --- | --- | --- | --- |
| Q14409 | GK3P | Putative glycerol kinase 3 | NS |
| O00764 | PDXK | Pyridoxal kinase | NS |
| P14618 | PKM | Pyruvate kinase PKM | NS |
| P54819 | AK2 | Adenylate kinase 2 | Mito Q/Fresh |
| Q96M32 | AK7 | Adenylate kinase 7 | NS |
| Q96MA6 | AK8 | Adenylate kinase 8 | NS |
| P00568 | AK1 | Adenylate kinase isoenzyme 1 | NS |
| Q86UN6 | AKAP14 | A-kinase anchor protein 14 | NS |
| O75969 | AKAP3 | A-kinase anchor protein 3 | NS |
| Q5JQC9 | AKAP4 | A-kinase anchor protein 4 | NS |
| P17858 | PFKL | ATP-dependent 6-phosphofructokinase, liver type | NS |
| P08237 | PFKM | ATP-dependent 6-phosphofructokinase, muscle type | NS |
| Q01813 | PFKP | ATP-dependent 6-phosphofructokinase, platelet type | NS |
| Q3LXA3 | DAK | Bifunctional ATP-dependent dihydroxyacetone kinase/FAD-AMP lyase (cyclizing);ATP-dependent dihydroxyacetone kinase;FAD-AMP lyase (cyclizing) | NS |
| Q13557 | CAMK2D | Calcium/calmodulin-dependent protein kinase type II subunit delta | NS |
| Q16566 | CAMK4 | Calcium/calmodulin-dependent protein kinase type IV | Mito-Gly/Fresh |
| O75952 | CABYR | Calcium-binding tyrosine phosphorylation-regulated protein | NS |
| P17612 | PRKACA | cAMP-dependent protein kinase catalytic subunit alpha | NS |
| P22612 | PRKACG | cAMP-dependent protein kinase catalytic subunit gamma | NS |
| P10644 | PRKAR1A | cAMP-dependent protein kinase type I-alpha regulatory subunit;cAMP-dependent protein kinase type I-alpha regulatory subunit, N-terminally processed | NS |
| P13861 | PRKAR2A | cAMP-dependent protein kinase type II-alpha regulatory subunit | NS |
| P48729 | CSNK1A1 | Casein kinase I isoform alpha | NS |
| P19784 | CSNK2A2 | Casein kinase II subunit alpha | NS |
| P68400 | CSNK2A1 | Casein kinase II subunit alpha;Casein kinase II subunit alpha 3 | NS |
| P67870 | CSNK2B | Casein kinase II subunit beta | NS |
| Q9Y259 | CHKB | Choline/ethanolamine kinase | Mito Q/Fresh  Glycerol/Fresh |
| O14578 | CIT | Citron Rho-interacting kinase | Glycerol/Fresh |
| P12277 | CKB | Creatine kinase B-type | NS |
| Q16854 | DGUOK | Deoxyguanosine kinase, mitochondrial | NS |
| P36507 | MAP2K2 | Dual specificity mitogen-activated protein kinase kinase 2 | NS |
| Q14410 | GK2 | Glycerol kinase 2 | NS |
| P49840 | GSK3A | Glycogen synthase kinase-3 alpha | NS |
| P19367 | HK1 | Hexokinase-1 | NS |
| O75569 | PRKRA | Interferon-inducible double-stranded RNA-dependent protein kinase activator A | NS |
| P29966 | MARCKS | Myristoylated alanine-rich C-kinase substrate | NS |
| Q9UJ70 | NAGK | N-acetyl-D-glucosamine kinase | NS |
| Q13232 | NME3 | Nucleoside diphosphate kinase 3 | NS |
| Q9Y5B8 | NME7 | Nucleoside diphosphate kinase 7 | NS |
| P15531 | NME1 | Nucleoside diphosphate kinase A | NS |
| P22392 | NME2 | Nucleoside diphosphate kinase B;Putative nucleoside diphosphate kinase | Glycerol/Fresh |
| P56597 | NME5 | Nucleoside diphosphate kinase homolog 5 | NS |
| Q8TBX8 | PIP4K2C | Phosphatidylinositol 5-phosphate 4-kinase type-2 gamma | NS |
| P00558 | PGK1 | Phosphoglycerate kinase 1 | NS |
| P07205 | PGK2 | Phosphoglycerate kinase 2 | NS |
| Q93100 | PHKB | Phosphorylase b kinase regulatory subunit beta | NS |
| P07311 | ACYP1 | Acylphosphatase-1 | NS |
| Q9UKK9 | NUDT5 | ADP-sugar pyrophosphatase | NS |
| P34913 | EPHX2 | Bifunctional epoxide hydrolase 2;Cytosolic epoxide hydrolase 2;Lipid-phosphate phosphatase | NS |
| P50583 | NUDT2 | Bis(5-nucleosyl)-tetraphosphatase [asymmetrical] | NS |
| Q9UHY7 | ENOPH1 | Enolase-phosphatase E1 | NS |
| P09467 | FBP1 | Fructose-1,6-bisphosphatase 1 | NS |
| Q15181 | PPA1 | Inorganic pyrophosphatase | NS |
| A0A024RBG1 | NUDT4 | Inositol monophosphatase 1 | NS |
| P29218 | IMPA1 | Inositol monophosphatase 1 | NS |
| P24666 | ACP1 | Low molecular weight phosphotyrosine protein phosphatase | NS |
| Q9UNW1 | MINPP1 | Multiple inositol polyphosphate phosphatase 1 | NS |
| A6NDG6 | PGP | Phosphoglycolate phosphatase | NS |
| Q9H008 | LHPP | Phospholysine phosphohistidine inorganic pyrophosphate phosphatase | NS |
| P15309 | ACPP | Prostatic acid phosphatase;PAPf39 | NS |
| Q7Z5V6 | PPP1R32 | Protein phosphatase 1 regulatory subunit 32 | NS |
| Q15435 | PPP1R7 | Protein phosphatase 1 regulatory subunit 7 | NS |
| P35813 | PPM1A | Protein phosphatase 1A | NS |
| Q9Y570 | PPME1 | Protein phosphatase methylesterase 1 | NS |
| O14990 | PPP1R2P9 | Putative type-1 protein phosphatase inhibitor 4 | NS |
| Q12913 | PTPRJ | Receptor-type tyrosine-protein phosphatase eta | NS |
| P10586 | PTPRF | Receptor-type tyrosine-protein phosphatase F | NS |
| Q13332 | PTPRS | Receptor-type tyrosine-protein phosphatase S | NS |
| P30153 | PPP2R1A | Serine/threonine-protein phosphatase 2A 65 kDa regulatory subunit A alpha isoform | NS |
| Q15257 | PPP2R4 | Serine/threonine-protein phosphatase 2A activator | NS |
| P67775 | PPP2CA | Serine/threonine-protein phosphatase 2A catalytic subunit alpha isoform | NS |
| Q08209 | PPP3CA | Serine/threonine-protein phosphatase 2B catalytic subunit alpha isoform | NS |
| P48454 | PPP3CC | Serine/threonine-protein phosphatase 2B catalytic subunit gamma isoform | NS |
| P60510 | PPP4C | Serine/threonine-protein phosphatase 4 catalytic subunit | NS |
| Q8TF05 | PPP4R1 | Serine/threonine-protein phosphatase 4 regulatory subunit 1 | NS |
| P53041 | PPP5C | Serine/threonine-protein phosphatase 5 | NS |
| O00743 | PPP6C | Serine/threonine-protein phosphatase 6 catalytic subunit | NS |
| O15084 | ANKRD28 | Serine/threonine-protein phosphatase 6 regulatory ankyrin repeat subunit A | NS |
| Q9UPN7 | PPP6R1 | Serine/threonine-protein phosphatase 6 regulatory subunit 1 | BM/Fresh  Mito Q/Fresh  Glycerol/Fresh  Mito-Gly/Fresh |
| O75170 | PPP6R2 | Serine/threonine-protein phosphatase 6 regulatory subunit 2 | BM/Fresh |
| Q9BRF8 | CPPED1 | Serine/threonine-protein phosphatase CPPED1 | NS |
| Q96HS1 | PGAM5 | Serine/threonine-protein phosphatase PGAM5, mitochondrial | NS |
| P62140 | PPP1CB | Serine/threonine-protein phosphatase PP1-beta catalytic subunit | NS |
| P36873 | PPP1CC | Serine/threonine-protein phosphatase PP1-gamma catalytic subunit | NS |
| O14829 | PPEF1 | Serine/threonine-protein phosphatase with EF-hands 1 | NS |
| Q9Y3F4 | STRAP | Serine-threonine kinase receptor-associated protein | NS |
| Q9BU02 | THTPA | Thiamine-triphosphatase | NS |
| Q9NRX4 | PHPT1 | 14 kDa phosphohistidine phosphatase | NS |
