# Supplementary material for: Unraveling Subcellular and Ultrastructural Changes During Vitrification of Human Spermatozoa: Effect of a Mitochondria-Targeted Antioxidant and a Permeable Cryoprotectant
Source: Front Cell Dev Biol. 2021 Jul 2;9:672862. doi: 10.3389/fcell.2021.672862 (PMC8284099; doi:10.3389/fcell.2021.672862)
Supplement: Supplementary file 12 [file Table_12.DOCX]

**Supplementary Table 16: list of heat shock proteins identified in human sperm and showing differentially altered proteins (DAPs) after vitrification**

| **Protein IDs** | **Gene names** | **Protein names** | **DAPs** |
| --- | --- | --- | --- |
| P61604 | HSPE1 | 10 kDa heat shock protein, mitochondrial | NS |
| P10809 | HSPD1 | 60 kDa heat shock protein, mitochondrial | NS |
| O95433 | AHSA1 | Activator of 90 kDa heat shock protein ATPase homolog 1 | NS |
| P48723 | HSPA13 | Heat shock 70 kDa protein 13 | NS |
| P0DMV9 | HSPA1B | Heat shock 70 kDa protein 1B | NS |
| P34931 | HSPA1L | Heat shock 70 kDa protein 1-like | NS |
| P34932 | HSPA4 | Heat shock 70 kDa protein 4 | NS |
| O95757 | HSPA4L | Heat shock 70 kDa protein 4L | NS |
| P11142 | HSPA8 | Heat shock cognate 71 kDa protein | NS |
| Q92598 | HSPH1 | Heat shock protein 105 kDa | NS |
| Q12931 | TRAP1 | Heat shock protein 75 kDa, mitochondrial | NS |
| P04792 | HSPB1 | Heat shock protein beta-1 | NS |
| P07900 | HSP90AA1 | Heat shock protein HSP 90-alpha | NS |
| P08238 | HSP90AB1 | Heat shock protein HSP 90-beta | NS |
| P54652 | HSPA2 | Heat shock-related 70 kDa protein 2 | NS |
| Q58FF8 | HSP90AB2P | Putative heat shock protein HSP 90-beta 2 | NS |
| P31689 | DNAJA1 | DnaJ homolog subfamily A member 1 | BM/Fresh |
| O60884 | DNAJA2 | DnaJ homolog subfamily A member 2 | NS |
| Q8WW22 | DNAJA4 | DnaJ homolog subfamily A member 4 | NS |
| P25685 | DNAJB1 | DnaJ homolog subfamily B member 1 | NS |
| Q9UBS4 | DNAJB11 | DnaJ homolog subfamily B member 11 | NS |
| P59910 | DNAJB13 | DnaJ homolog subfamily B member 13 | NS |
| O75190 | DNAJB6 | DnaJ homolog subfamily B member 6 | NS |
| Q8NHS0 | DNAJB8 | DnaJ homolog subfamily B member 8 | NS |
| Q9UBS3 | DNAJB9 | DnaJ homolog subfamily B member 9 | NS |
| Q13217 | DNAJC3 | DnaJ homolog subfamily C member 3 | NS |
| Q9H3Z4 | DNAJC5 | DnaJ homolog subfamily C member 5 | NS |
