# Supplementary material for: Unraveling Subcellular and Ultrastructural Changes During Vitrification of Human Spermatozoa: Effect of a Mitochondria-Targeted Antioxidant and a Permeable Cryoprotectant
Source: Front Cell Dev Biol. 2021 Jul 2;9:672862. doi: 10.3389/fcell.2021.672862 (PMC8284099; doi:10.3389/fcell.2021.672862)
Supplement: Supplementary file 13 [file Table_13.DOCX]

**Supplementary Table 17: List of candidates proteins identified responsible for sperm-egg fusion and fertilization but not affected by vitrification process**

| **Protein IDs** | **Gene names** | **Protein names** | **DAPs** |
| --- | --- | --- | --- |
| Q8IYV9 | IZUMO1 | Izumo sperm-egg fusion protein 1 | NS |
| Q6UXV1 | IZUMO2 | Izumo sperm-egg fusion protein 2 | NS |
| Q1ZYL8 | IZUMO4 | Izumo sperm-egg fusion protein 4 | NS |
| Q6UWM5 | GLIPR1L1 | GLIPR1-like protein 1 | NS |
| P12830 | CDH1 | Cadherin-1;E-Cad/CTF1;E-Cad/CTF2;E-Cad/CTF3 | NS |
| P07237 | P4HB | Protein disulfide-isomerase | NS |
| P30101 | PDIA3 | Protein disulfide-isomerase A3 | NS |
| P13667 | PDIA4 | Protein disulfide-isomerase A4 | NS |
| Q15084 | PDIA6 | Protein disulfide-isomerase A6 | NS |
| Q8N807 | PDILT | Protein disulfide-isomerase-like protein of the testis | NS |
| P61626 | LYZ | Lysozyme C | NS |
| Q6UWQ5 | LYZL1 | Lysozyme-like protein 1 | NS |
| Q7Z4W2 | LYZL2 | Lysozyme-like protein 2 | NS |
| Q96KX0 | LYZL4 | Lysozyme-like protein 4 | NS |
| O75951 | LYZL6 | Lysozyme-like protein 6 | NS |
| Q9UHI8 | ADAMTS1 | A disintegrin and metalloproteinase with thrombospondin motifs 1 | NS |
| O14672 | ADAM10 | Disintegrin and metalloproteinase domain-containing protein 10 | NS |
| Q8TC27 | ADAM32 | Disintegrin and metalloproteinase domain-containing protein 32 | NS |
| Q9H2U9 | ADAM7 | Disintegrin and metalloproteinase domain-containing protein 7 | NS |
| Q13443 | ADAM9 | Disintegrin and metalloproteinase domain-containing protein 9 | NS |
| Q9UHI8 | ADAMTS1 | A disintegrin and metalloproteinase with thrombospondin motifs 1 | NS |
| O14967 | CLGN | Calmegin | NS |
| P27797 | CALR | Calreticulin | NS |
| P38567 | SPAM1 | Hyaluronidase PH-20 | NS |
| Q969V4 | TEKT1 | Tektin-1 | NS |
| Q9UIF3 | TEKT2 | Tektin-2 | NS |
| Q9BXF9 | TEKT3 | Tektin-3 | NS |
| Q8WW24 | TEKT4 | Tektin-4 | NS |
| Q96M29 | TEKT5 | Tektin-5 | NS |
| Q9BS86 | ZPBP | Zona pellucida-binding protein 1 | NS |
| Q6X784 | ZPBP2 | Zona pellucida-binding protein 2 | NS |
| P00441 | SOD1 | Superoxide dismutase [Cu-Zn] | NS |
| P04179 | SOD2 | Superoxide dismutase [Mn], mitochondrial | NS |
| P04040 | CAT | Catalase | NS |
