# Supplementary material for: Unraveling Subcellular and Ultrastructural Changes During Vitrification of Human Spermatozoa: Effect of a Mitochondria-Targeted Antioxidant and a Permeable Cryoprotectant
Source: Front Cell Dev Biol. 2021 Jul 2;9:672862. doi: 10.3389/fcell.2021.672862 (PMC8284099; doi:10.3389/fcell.2021.672862)
Supplement: Supplementary file 14 [file Image_1.PDF]

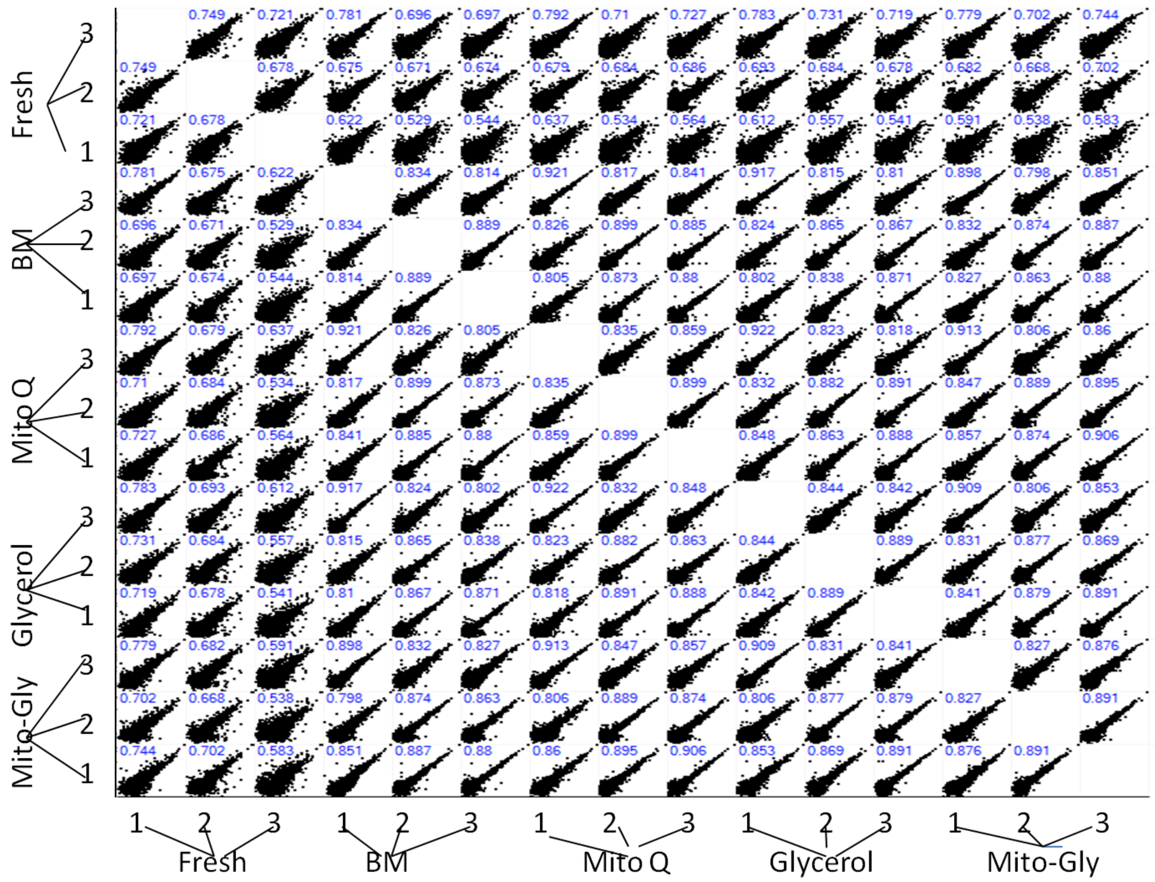

Supplementary Figure 1: Multi-scatter plots with Pearson correlation values of 0.5–1.0 suggest a good correlation between data obtained from the different experimental groups and biological replicates. Numbers on each panel represent correlation coefficient (R) values. Pearson correlations were calculated though the default ‘multiscatter’ graphical functions in Perseus.
